# Single-cell profiling reveals the importance of CXCL13/CXCR5 axis biology in lymphocyte-rich classic Hodgkin lymphoma

**DOI:** 10.1073/pnas.2105822118

**Published:** 2021-10-06

**Authors:** Tomohiro Aoki, Lauren C. Chong, Katsuyoshi Takata, Katy Milne, Ashley Marshall, Elizabeth A. Chavez, Tomoko Miyata-Takata, Susana Ben-Neriah, Doria Unrau, Adele Telenius, Merrill Boyle, Andrew P. Weng, Kerry J. Savage, David W. Scott, Pedro Farinha, Sohrab P. Shah, Brad H. Nelson, Christian Steidl

**Affiliations:** ^a^Centre for Lymphoid Cancer, British Columbia (BC) Cancer, Vancouver, BC, Canada V5Z 1L3;; ^b^Department of Pathology and Laboratory Medicine, University of British Columbia, Vancouver, BC, Canada V6T 1Z7;; ^c^Division of Molecular and Cellular Pathology, Niigata University Graduate School of Medical and Dental Sciences, 951-8510 Niigata, Japan;; ^d^Deeley Research Centre, BC Cancer, Victoria, BC, Canada V8R 6V5;; ^e^Department of Biochemistry and Microbiology, University of Victoria, Victoria, BC, Canada;; ^f^Terry Fox Laboratory, BC Cancer, Vancouver, BC, Canada V5Z 1L3;; ^g^Department of Molecular Oncology, BC Cancer, Vancouver, BC, Canada V5Z 1L3;; ^h^Department of Epidemiology and Biostatistics, Memorial Sloan Kettering Cancer Center, New York, NY 10017;; ^i^Department of Medical Genetics, University of British Columbia, Vancouver, BC, Canada V6T 1Z4

**Keywords:** Hodgkin lymphoma, single-cell analyses, CXCL13, PD-1, PD-L1

## Abstract

Lymphocyte-rich classic Hodgkin lymphoma (LR-CHL) is a rare subtype of Hodgkin lymphoma. Recent technical advances have allowed for the characterization of specific cross-talk mechanisms between malignant Hodgkin Reed-Sternberg (HRS) cells and different normal immune cells in the tumor microenvironment (TME) of CHL. However, the TME of LR-CHL has not yet been characterized at single-cell resolution. Here, using single-cell RNA sequencing (scRNA-seq), we examined the immune cell profile of 8 cell suspension samples of LR-CHL in comparison to 20 samples of the mixed cellularity (MC, 9 cases) and nodular sclerosis (NS, 11 cases) subtypes of CHL, as well as 5 reactive lymph node controls. We also performed multicolor immunofluorescence (MC-IF) on tissue microarrays from the same patients and an independent validation cohort of 31 pretreatment LR-CHL samples. ScRNA-seq analysis identified a unique CD4^+^ helper T cell subset in LR-CHL characterized by high expression of Chemokine C-X-C motif ligand 13 (CXCL13) and PD-1. PD-1^+^CXCL13^+^ T cells were significantly enriched in LR-CHL compared to other CHL subtypes, and spatial analyses revealed that in 46% of the LR-CHL cases these cells formed rosettes surrounding HRS cells. MC-IF analysis revealed CXCR5^+^ normal B cells in close proximity to CXCL13^+^ T cells at significantly higher levels in LR-CHL. Moreover, the abundance of PD-1^+^CXCL13^+^ T cells in the TME was significantly associated with shorter progression-free survival in LR-CHL (*P* = 0.032). Taken together, our findings strongly suggest the pathogenic importance of the CXCL13/CXCR5 axis and PD-1^+^CXCL13^+^ T cells as a treatment target in LR-CHL.

## Significance

Our study provides detailed functional and spatial characteristics of immune cells in the LR-CHL microenvironment at single-cell resolution. We describe detailed T cell subset definitions and importantly identified a unique CD4^+^PD-1^+^CXCL13^+^CXCR5^−^ TFH-like subset that surrounds HRS cells, appears in close proximity to CXCR5^+^ B cells, and is associated with poor clinical outcome. We also uncovered unique PD-1/PD-L1 axis biology in LR-CHL, namely a negative correlation between PD-L1 genetic alterations on HRS cells and PD-1 protein expression in the tumor microenvironment. Importantly, our findings contribute to a deeper understanding of cellular cross-talk in LR-CHL, which may aid in the development of novel biomarkers and targeted treatment strategies.

Classic Hodgkin lymphoma (CHL) is a subtype of B cell lymphoma that is uniquely characterized by cross-talk of malignant cells with different types of noncancerous normal immune cells in the tumor microenvironment (TME). On the basis of the morphology and immunophenotype of the malignant Hodgkin and Reed-Sternberg (HRS) cells, infiltrating immune cells and fibroblastic elements, four histological subtypes of CHL are recognized: nodular sclerosis (NS), mixed cellularity (MC), lymphocyte rich (LR), and lymphocyte depleted (LD) (1). Lymphocyte-rich CHL (LR-CHL) is a rare subtype of Hodgkin lymphoma, which accounts for ∼5% of all CHL. The disease is more common in elderly males and exhibits less frequent mediastinal involvement and bulky disease when compared to other CHL subtypes (2–4). Histologically, LR-CHL is characterized by a predominant nodular pattern with few scattered HRS cells distributed in T cell–rich zones, with numerous small lymphocytes and an absence of eosinophils and neutrophils in the nodules (2). Clinically, patients often present with localized peripheral lymphadenopathy and it typically is associated with a favorable outcome (3).

In CHL, nonmalignant immune cell populations make up more than 99% of the tumor bulk and create a tumor-supportive milieu via cross-talk with the rare HRS cells (∼1%) (1, 5). The presence of specific immune cell types, including macrophages and T cells, as well as their spatial arrangement, plays a fundamental role in creating an immunosuppressive microenvironment in CHL. The presence of these immune cell types has been shown to have prognostic value and highlights the dependency of HRS cells on the TME for survival and immune evasion (6–11). CD4^+^ T cells are significantly enriched in CHL compared to reactive lymphadenopathies, which is consistent with previous literature that showed more frequent major histocompatibility class II (MHC-II) expression on HRS cells than MHC-I (12, 13). Of note, loss of MHC-II expression on HRS cells was found to be associated with inferior response to immune checkpoint inhibitors in CHL (12, 14). This indicates the importance of CD4^+^ T cells in CHL pathogenesis, and suggests MHC-II/CD4-dependent interactions between malignant cells and the TME.

Recent technical advances, including single-cell sequencing and spatial imaging analysis, revealed a high abundance of various types of immunosuppressive CD4^+^ T cells in the TME of CHL. These expressed coinhibitory receptors, including LAG3 and CTLA4 (15, 16). The interactions between these receptors and their ligands are believed to be the driving force behind the impaired immune response and unique microenvironment composition in CHL. Interestingly, despite the high efficacy of anti–PD-1 blockade in relapsed/refractory CHL (17–22), PD-1^+^ cells are not particularly abundant in Hodgkin lymphoma (HL) tissue except in LR-CHL (15, 23). PD-1^+^ T cells forming rosettes around HRS cells are reported to be present in approximately half of LR-CHL cases (2). However, the specific role of these PD-1^+^ T cells, their coexpression patterns with other coinhibitory receptors, and the overall TME composition, has not been well characterized in LR-CHL due to disease rarity.

Here, using single-cell RNA sequencing (scRNA-seq) and multicolor immunofluorescence (MC-IF), we identified LR-CHL–enriched immune cell subsets, including CXCL13^+^ T follicular helper (TFH)-like cells that were shown to be surrounding HRS cells in spatial analysis and were in close contact with CXCR5^+^ B cells. On the strength of an unprecedented number of single-cell transcriptomes in combination with multiplexed spatial assessment, we deciphered the unique immune cell architecture of the TME in LR-CHL with implications for previously uncharacterized treatment targets.

## Results

### The LR-CHL–Specific Immune Microenvironment at Single-Cell Resolution.

**Fig. 1.**
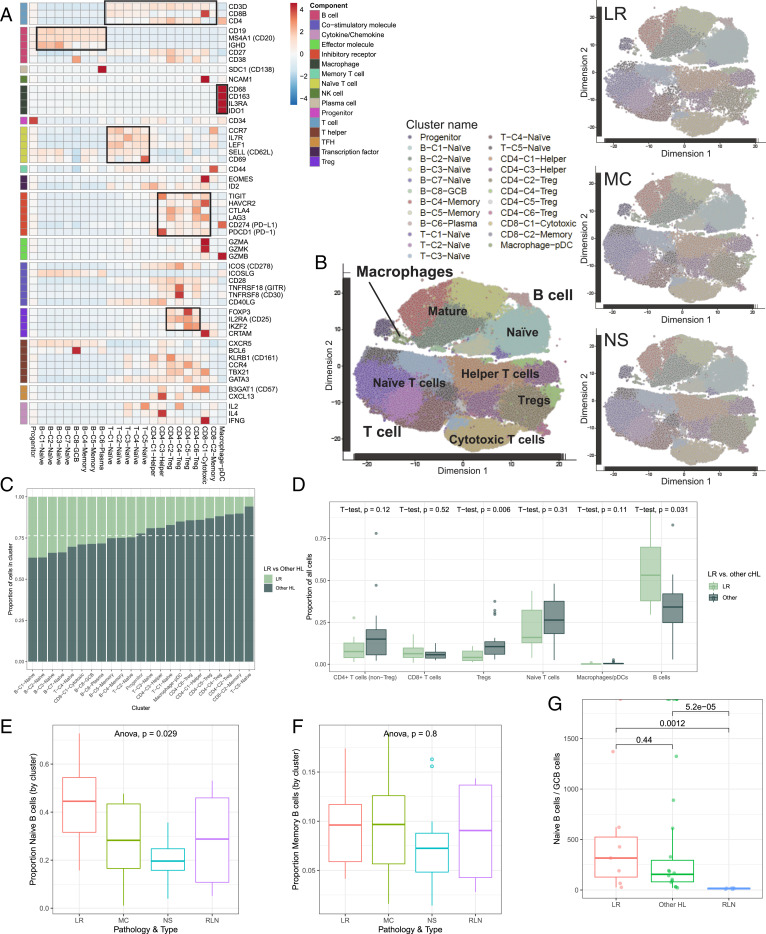
Immune cell atlas of the LR-CHL microenvironment at single-cell resolution. Cells from 28 CHL and 5 RLN cases were clustered using the PhenoGraph algorithm to identify groups of cells with similar expression patterns. (*A*) Heatmap summarizing mean expression (normalized and log transformed) of selected canonical markers in each cluster. Data have been scaled row-wise for visualization. The covariate bar on the *Left* side indicates the component associated with each gene, and black boxes highlight prominent expression of known subtype genes. (*B*) Single-cell expression of all cells from CHL and RLN in tSNE space (first two dimensions). Cells are colored according to PhenoGraph cluster. Subsets of cells from each CHL subtype are shown on the same coordinates. (*C*) Proportion of cells in each cluster originating from LR-CHL (light green) and other CHL (dark green) samples. The dashed white line represents the total proportion of cells from other CHL samples in the merged population. (*D*) The proportion of cells assigned to a given immune cell type (as determined by cluster annotation) was calculated for each sample. Boxplots summarize the distribution of the proportions for all samples, grouped by pathological subtype (LR-CHL or other CHL subtype). *P* values are shown *Above* and demonstrate a significant increase in the proportion of B cells present in LR-CHL compared to other CHL. (*E* and *F*) Boxplots summarizing the proportion of naïve (*E*) and memory (*F*) B cells relative to total cells in each sample, separated according to CHL subtype and RLNs. (*G*) Ratio of naïve B cells/germinal center B cells (by cluster assignment) according to pathological subtype. *P* values were calculated using *t* tests.

To investigate the specific immune cell profile of the LR-CHL TME, we utilized our previously published scRNA-seq cohort of CHL and sequenced an additional 7 LR-CHL cases for comparison (15). The resulting cohort contained data from 28 CHL patients, including 8 LR, 11 NS, and 9 MC, plus 5 reactive lymph nodes (RLNs) sequenced as normal comparators (*SI Appendix*, Tables S1 and S2). In total, transcriptomes were generated for 146,473 sorted live cells (*SI Appendix*, Table S3). After batch correction and normalization (*Materials and Methods*), unsupervised clustering of the single-cell expression profiles yielded a total of 23 clusters. We assigned each cluster to a cell type based on the expression of genes described in published transcriptome data of sorted immune cells (24) and known canonical markers (Fig. 1 *A* and *B* and *SI Appendix*, Fig. S1 and Dataset S1). This produced 13 T cell clusters, 8 B cell clusters, 1 macrophage/ plasmacytoid dendritic cell (pDC) cluster, and 1 progenitor cell cluster. Notably, we did not observe any clusters resembling HRS cells, likely due to size limitations in the microfluidics device or loss of HRS cells during the freezing and thawing process. While most immune cell phenotypes exhibited overlap among pathological subtypes, as demonstrated by clusters containing a mixture of cell types, we observed an enrichment of cells from LR-CHL in some specific cell clusters (Fig. 1 *B* and *C*). Of interest, regulatory T cells (Tregs), which we and others have observed as an enriched immune cell type in CHL (9, 15, 25, 26), were significantly decreased in LR-CHL compared to other CHL subtypes (*P* = 0.006, *t* test; Fig. 1*D*). All 4 Treg clusters, including those characterized by high LAG3 expression and those with high FOXP3 expression, had a low proportion of cells originating from LR samples, suggesting a relative paucity of Tregs as a unique feature of the LR-CHL TME (Fig. 1 *C* and *D* and *SI Appendix*, Fig. S2 *A* and *B*). Conversely, we found that B cell clusters were uniquely enriched in LR-CHL cells when compared to other CHL subtypes, and specifically all 4 naïve B cell clusters were dominated by cells derived from LR tumors (Fig. 1 *C–E*). While the proportion of cells assigned to naïve B cell clusters was significantly higher in LR-CHL samples compared to other CHL subtypes and RLNs, the proportion of memory B cells was comparable (Fig. 1*F*). We confirmed B cell enrichment in LR-CHL on the protein level by flow cytometry (*SI Appendix*, Fig. S2*C*). Intriguingly, the proportion of cells assigned to the germinal center B cell (GCB) cluster was significantly lower in LR-CHL compared to RLN samples (Fig. 1*G* and *SI Appendix*, Fig. S2*D*).

### Single-Cell Expression Patterns of LR-CHL–Specific Immune Subsets.

**Fig. 2.**
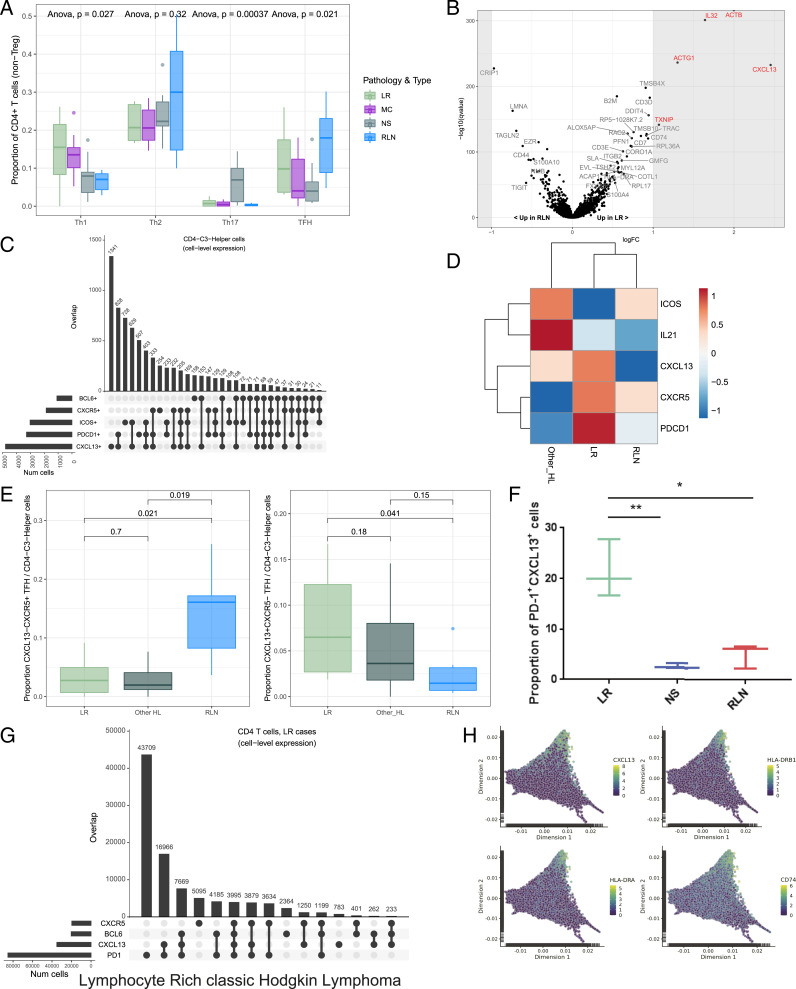
Detailed characterization and coexpression patterns of helper T cells in the tumor microenvironment of LR-CHL. (*A*) The proportion of helper T cells assigned to various cell subsets was calculated for each sample (see *SI Appendix*, *Materials and Methods* for assignment criteria). Boxplots summarize the distribution of the proportions for all samples, grouped by pathological subtype. *P* values, calculated using an Anova test, are shown *Above*. (*B*) Volcano plot showing differentially expressed genes between cells in the TFH cell cluster (CD4-C3-Helper) originating from LR-CHL vs. RLN samples. The *y* axis summarizes *P* values corrected for multiple testing using the Benjamini–Hochberg method (“q values”). Significant genes are labeled in red (q value <0.05 and absolute log2 fold change ≥1). (*C*) UpSet plot showing coexpression patterns of inhibitory receptors (CXCR5, PDCD1 [PD-1], CXCL13, ICOS, and BCL6) for individual cells in the TFH cluster. (*D*) Heatmap showing mean expression of TFH markers for cells in the CD4-C3-Helper cluster across all samples, grouped by pathological subtype. Expression values have been scaled row-wise for visualization. (*E*) Boxplots summarizing the proportion of classical TFH (*Left*) and CXCL13^+^ helper T cells (*Right*) in each sample, separated according to pathological subtype. *P* values, calculated with *t* tests, are shown *Above*. (*F*) Boxplot summarizing the proportion of PD-1^+^CXCL13^+^ cells from each cell suspension sample analyzed by flow cytometry, separated according to pathological subtype. Data are shown as the mean ± SEM (*n* = 3). *P < 0.05; **P < 0.01. (*G*) UpSet plot showing coexpression patterns on CD4^+^ T cells in LR-CHL by flow cytometry. (*H*) Cellular trajectories were inferred using diffusion map analysis of cells in CD4^+^ helper T cell clusters. Individual cells are shown in the first two resulting dimensions. Expression levels are shown for the four genes most positively correlated with dimension 2 score (*SI Appendix*, *Materials and Methods*).

TFH cells play an important role in normal B cell development by supporting B cell differentiation and antibody production (27, 28). Our data demonstrated preferential enrichment of the TFH population in LR-CHL as compared to other CHLs (Fig. 2*A*). To investigate the characteristics of TFH cells in LR-CHL, we performed differential gene expression analysis between cells from LR-CHL and RLN samples in the cluster that most resembled a TFH profile (“CD4-C3-Helper”). Of note, CXCL13 was identified as the most up-regulated gene in LR-derived TFH cells compared to RLNs (Fig. 2*B*). CXCL13, which is the canonical ligand of CXCR5, is well known as a B cell attractant that works via the CXCL13/CXCR5 signaling axis and is highly expressed on follicular dendritic cells (FDCs) in germinal center lesions (29). Analyzing coexpression patterns on the single-cell level revealed that the majority of CXCL13^+^ T cells coexpressed PD-1 and ICOS, which are known as universal TFH markers, but coexpression with CXCR5, another common TFH marker, was rarely observed (Fig. 2*C*). Notably, the coexpression pattern of TFH markers was variable among disease subtypes, suggesting a potentially distinct role of TFH cells in each subtype (Fig. 2*D*). The proportion of TFH cells with a classical TFH profile coexpressing CXCR5 and PD-1 was high in RLNs, whereas TFH cells coexpressing PD-1 and CXCL13, but not CXCR5, were significantly more prevalent in LR-CHL (Fig. 2*E* and *SI Appendix*, Fig. S3). TFH coexpression patterns in LR-CHL were validated on the protein level by flow cytometry (FCM) using cell suspensions from primary CHL patients (*n* = 3) and RLN samples (*n* = 3) gated for CD4^+^ T cells (Fig. 2 *F* and *G* and *SI Appendix*, Fig. S4). We confirmed that PD-1^+^CXCL13^+^CD4^+^ T cells were significantly enriched in LR-CHL compared to both RLN and NS samples on the protein level (Fig. 2*F*). Intriguingly, differential expression between classical CXCR5^+^CXCL13^−^ TFH cells and CXCL13^+^CXCR5^−^ TFH-like cells revealed higher expression of MHC-II genes in the CXCL13^+^CXCR5^−^ population (*SI Appendix*, Fig. S5). MHC-II expression is a known marker of T cell activation (30), indicating that the CXCL13^+^CXCR5^−^ population exhibits an activated phenotype.

To explore the functional role of CXCL13^+^ T cells, we next applied the diffusion map algorithm (31, 32) with the aim of characterizing differentiation states among helper T cells (Fig. 2*H*). CXCL13^+^ cells were enriched at the positive end of the second dimension, which was correlated with expression of genes representative of a terminal differentiation signature (*SI Appendix*, Fig. S6). The other most positively correlated genes tracking with dimension 2 were MHC-II genes, providing further evidence for an activated phenotype in the CXCL13^+^ T helper cells.

### Spatial Assessment of CXCL13^+^ T Cells and HRS Cells.

**Fig. 3.**
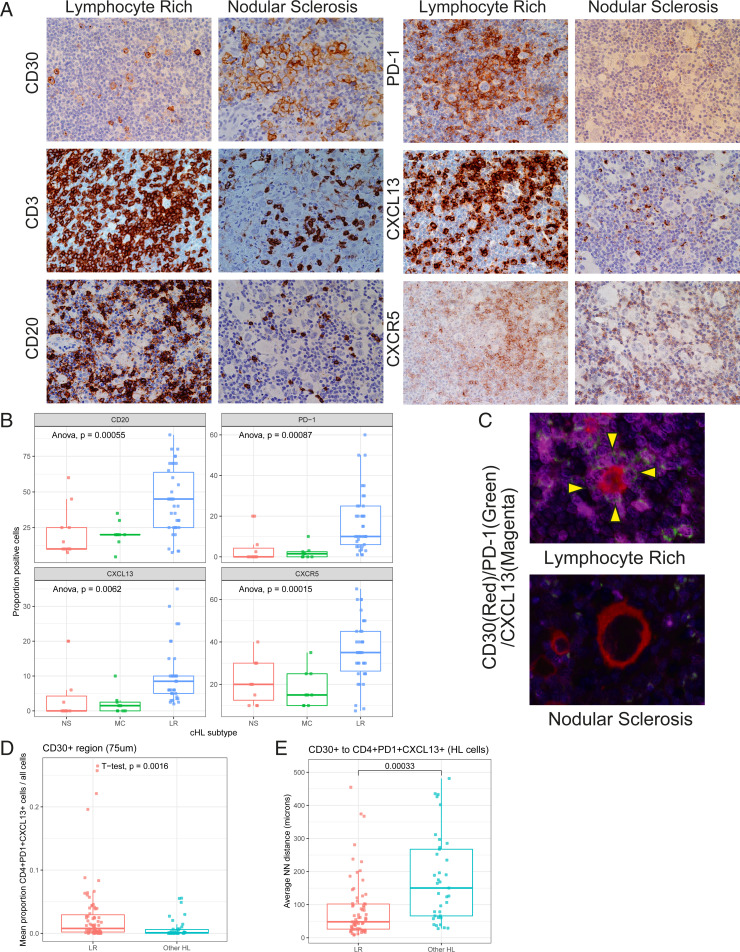
Spatial distribution of HRS cells and CXCL13^+^ T cells in LR-CHL. (*A*) IHC staining for major immune cell markers in representative cases with either LR-CHL (*Left*; LRCHL20) or nodular sclerosis CHL (*Right*; CHL03) (×400). (*B*) Boxplot showing proportions of positive cells by IHC for major immune cell markers according to disease subtype. *P* values were calculated using Anova tests. (*C*) Multicolor IF staining (CHL05 and LRCHL16) for CD30 (red), PD-1 (green), and CXCL13 (magenta) shows localization of CD4^+^PD-1^+^CXCL13^+^ T cells in rosettes around HRS cells in cases with LR-CHL. No rosettes are observed in cases of other CHL subtypes (e.g., nodular sclerosis shown here). (*D*) Boxplot showing the proportion of CD4^+^PD-1^+^CXCL13^+^ T cells in the region surrounding CD30^+^ cells (HRS) for each sample, separated by CHL subtype. The surrounding region was defined by a distance of 75 μm. (*E*) Average nearest neighbor (NN) distance from an HRS cell (defined by CD30^+^) to its closest CD4^+^PD-1^+^CXCL13^+^ cell was calculated per sample and plotted by pathological subtype. *P* values were calculated using *t* tests.

We next sought to validate our scRNA-seq findings in histologically intact tissue sections and understand the spatial relationship between CXCL13^+^ T cells and malignant HRS cells. We created a tissue microarray (TMA) from tumor tissue of 37 LR-CHL patients, which included 6 cases from our scRNA-seq cohort (*SI Appendix*, Table S4), and performed immunohistochemistry (IHC) on this TMA and the TMA from our previous scRNA-seq cohort (*n* = 26; 1 LR, 9 MC, 11 NS, 5 RLN) (15). IHC revealed that CXCL13^+^ T cells were significantly enriched in the LR-CHL TME compared to other subtypes (Fig. 3 *A* and *B*). Approximately half of the LR-CHL cases (46%) showed CXCL13^+^ T cells surrounding HRS cells (“rosettes”), whereas only 13% of patients with other CHL subtypes showed CXCL13^+^ T cell rosettes. Since PD-1^+^ T cell rosettes have been previously described as a specific feature of LR-CHL (2), we next evaluated PD-1 IHC on the CHL TMAs. Of note, all LR-CHL cases with CXCL13^+^ T cell rosettes also showed PD-1^+^ cell rosettes surrounding HRS cells, and PD-1^+^ cells were also significantly enriched in the LR-CHL TME compared to other CHLs. Consistent with scRNA-seq data, we also observed that CD20^+^ B cells were significantly enriched in LR-CHL (Fig. 3*B*). To validate coexpression patterns on the CXCL13^+^ T cells, we applied MC-IF on the same TMAs. We confirmed that most CD4^+^CXCL13^+^ T cells coexpressed PD-1, and the proportion of CD4^+^PD-1^+^CXCL13^+^ T cells in HRS-surrounding regions (i.e., within 75 μm of a CD30^+^ cell) was significantly increased in LR-CHL (Fig. 3 *C* and *D*). Similarly, the average distance between CD30^+^ cells (HRS cells) and their nearest CD4^+^PD-1^+^CXCL13^+^ T cell was significantly shorter in LR-CHL (Fig. 3*E*).

### CXCL13/CXCR5 Interaction in LR-CHL.

**Fig. 4.**
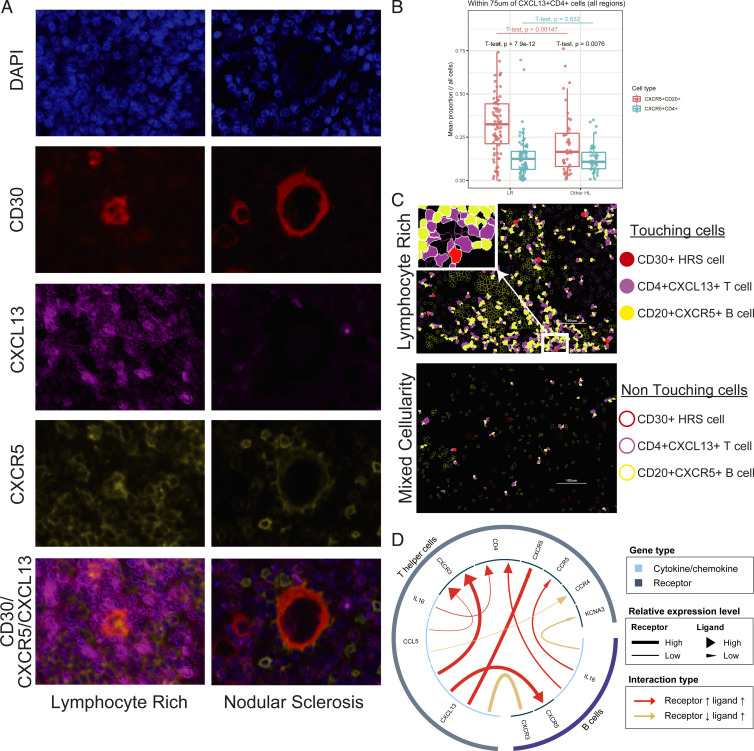
CXCL13/CXCR5 interaction in LR-CHL. (*A*) Multicolor IF staining (CHL05 and LRCHL16) for CD30 (red), CXCL13 (magenta), and CXCR5 (yellow), shows localization of CXCR5^+^ cells near CXCL13^+^ cells in the region surrounding HRS cells in cases with LR-CHL. CXCL13^+^ cells (magenta) are rarely coexpressed with CXCR5 (yellow). (*B*) Boxplot showing the proportion of CD20^+^CXCR5^+^ B cells and CD4^+^CXCR5^+^ T cells in the region surrounding CD4^+^CXCL13^+^ T cells (within 75 μm) for each sample, separated by pathological subtype. *t* tests show comparisons both within the subtypes (LR or other HL) and across subtypes (LR vs. other HL). (*C*) Membrane map depicting CD4^+^CXCL13^+^ T cells (magenta), CD20^+^CXCR5^+^ B cells (yellow), and CD30^+^ HRS cells (red). Touching cells (CD30^+^ HRS cells/CD4^+^CXCL13^+^ T cells and CD4^+^CXCL13^+^ T cells/CD20^+^CXCR5^+^ B cells) are represented by filled shapes. (*D*) An enriched positive interaction between CXCL13 on T helper cells and CXCR5 on B cells in LR-CHL was predicted using the iTALK tool.

As CXCR5 is the primary receptor for CXCL13, we next investigated CXCR5^+^ cells in the TME of LR-CHL with the aim of characterizing their relationship with CD4^+^CXCL13^+^ T cells. MC-IF analysis revealed that the majority of CXCR5^+^ cells in the TME were B cells (CD20^+^) (*SI Appendix*, Fig. S7). In contrast to CD4^+^CXCR5^+^ T cells, CD20^+^CXCR5^+^ B cells were significantly enriched in regions surrounding CD4^+^CXCL13^+^ T cells (Fig. 4 *A* and *B*). Notably, CXCL13^+^ cells rarely coexpressed CXCR5, confirming a mostly mutually exclusive pattern between CXCR5 and CXCL13 in the TME of LR-CHL (Fig. 4*A*). Furthermore, the proportion of CD20^+^CXCR5^+^ cells in regions surrounding CD4^+^CXCL13^+^ T cells was significantly increased in LR-CHL when compared with other CHL subtypes, while the proportion of CD4^+^CXCR5^+^ cells was comparable between subtypes (Fig. 4 *B* and *C*). The iTALK tool (33) was used to predict receptor/ligand interactions enriched in LR-CHL compared to other CHLs and confirmed a significantly increased positive interaction between CXCL13 on helper T cells and CXCR5 on B cells (Fig. 4*D*), supporting the importance of the CXCR5/CXCL13 axis in the specific pathogenesis of LR-CHL. In contrast, TFH cells in a normal RLN germinal center showed a typical TFH cell phenotype (CXCR5^+^CXCL13^−^) (*SI Appendix*, Fig. S8).

### PD-1/PD-L1 Biology in LR-CHL Patients.

**Fig. 5.**
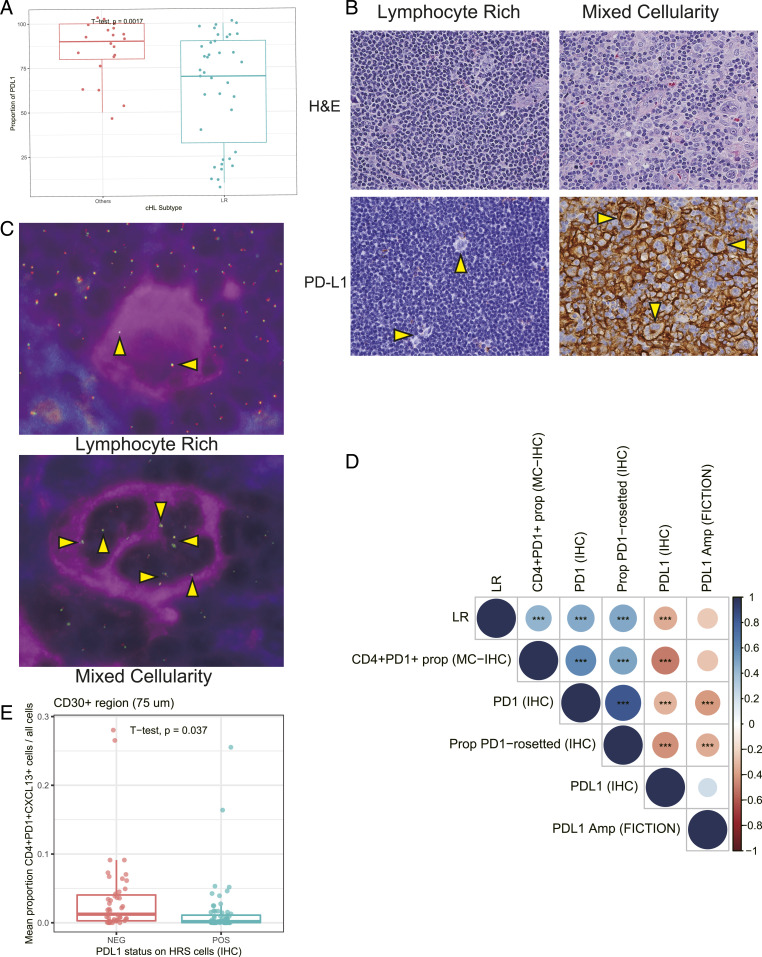
PD-L1 genomic alterations in HRS cells in LR-CHL. (*A*) Boxplot summarizing the proportion of PD-L1^+^ HRS cells by IHC in each sample, separated according to CHL subtype. (*B*) IHC staining for PD-L1 in representative CHL cases (×400; CHL20 and CHL25). (*C*) Combined immunofluorescence for CD30 (magenta) and fluorescence in situ hybridization (FISH) using bacterial artificial chromosome probes in the *PD-L1* and *PD-L2* region (green and red signals) shows *PD-L1/L2* amplification in HRS cells in mixed cellularity CHL (*Lower*) (×400; CHL20) but not in lymphocyte-rich CHL (*Upper*) (×400; LRCHL01). Of 26 LR-CHL cases, 5 (19%) cases showed *PD-L1/L2* amplification in HRS cells. (*D*) Dotplot showing correlation of *PD-L1* alteration status in HRS cells with expression level of major immune cell markers (IHC). Dot size and color summarize Pearson correlation values, with positive correlations represented in blue and negative correlations represented in red. Asterisks represent associated *P* values (***P < 0.001). (*E*) Boxplot showing the proportion of CD4^+^PD-1^+^CXCL13^+^ T cells in the region surrounding CD30^+^ cells (HRS) for each sample, separated by PD-L1 expression status on HRS cells (IHC). Of the 58 CHL samples, 34 cases (59%) showed high PD-L1 expression on HRS cells. The surrounding region was defined by a distance of 75 μm.

To investigate PD-1/PD-L1 biology in LR-CHL, we next investigated the expression of PD-L1 on HRS cells. HRS cells often exhibit overexpression of PD-L1 through copy number gains and amplifications of the 9p24.1 locus where its coding gene (*CD274*) resides (34–38). Surprisingly, regardless of the proportion of PD-1^+^ T cells, PD-L1 expression on HRS cells was significantly lower in LR-CHL when compared with other CHL subtypes (Fig. 5 *A* and *B*). Furthermore, we also performed copy number analysis of the *CD274* and *PDCD1LG2* (encoding PD-L2) genes in HRS cells using the FICTION technique (36), which enables quantitative assessment of the copy number of the *CD274/PDCD1LG2* genes in CD30-labeled IHC sections on a TMA (Fig. 5*C*). Interestingly, LR-CHL cases showed fewer copy number amplifications of *CD274/PDCD1LG2* (19%, 5/26 cases) compared to other CHL subtypes (43%, 9/21 cases), and *CD274* copy number amplification status was positively correlated with PD-L1 protein expression on HRS cells (Fig. 5*D*). Of note, PD-L1 expression status of HRS cells was negatively correlated with the proportion of PD-1 rosettes in the TME, and CD4^+^PD-1^+^CXCL13^+^ T cells in the region surrounding HRS cells were significantly fewer in cases with PD-L1^+^ HRS cells (*n* = 34) (Fig. 5*E*). This might indicate a potential negative regulation and depletion of PD-1^+^ T cells if exposed to PD-L1^+^ HRS cells. Taken together these results suggest distinct PD-1/PD-L1–related biology in LR-CHL when compared to other CHL subtypes.

### TGF-β Induces CXCL13^+^ T Cells.

**Fig. 6.**
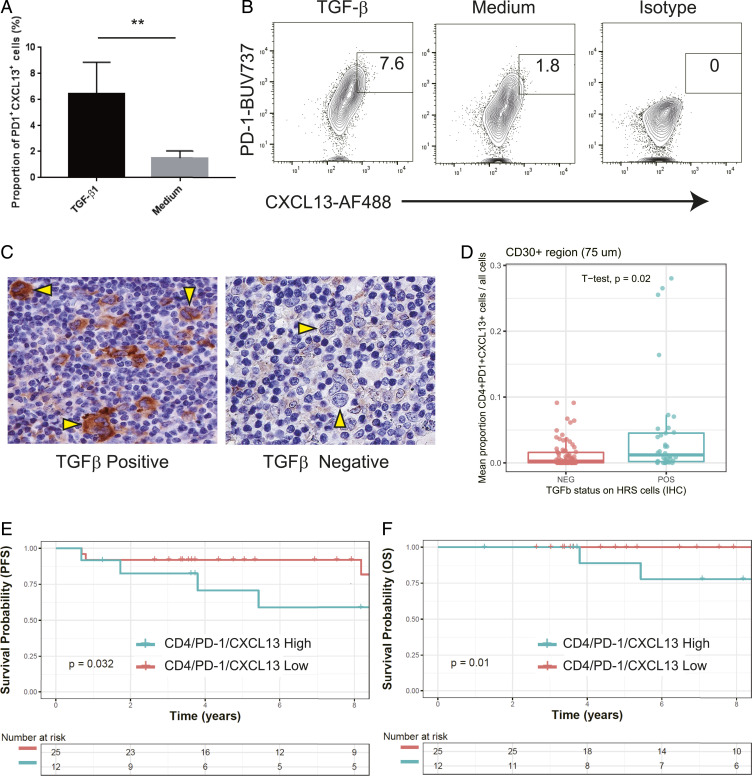
TGF-β induces a PD-1^+^CXCL13^+^ T cell population. (*A*) The proportion of PD-1^+^CXCL13^+^ cells among CD4^+^ T cells isolated from PBMCs after coculture with TGF-β or medium only. Data are shown as the mean ± SEM (*n* = 5) (**P < 0.01). (*B*) Representative flow cytometric analysis of PD-1 and CXCL13 expression on CD4^+^ T cells isolated from PBMCs cultured with TGF-β (*Left*), medium (*Middle*), or isotype control (*Right*). (*C*) IHC staining for TGF-β in representative cases with either positive (*Left*) or negative (*Right*) HRS cells (×400; CHL19 and LRCHL010). Of the 58 CHL cases, 18 cases (31%) showed high TGF-β expression on HRS cells. (*D*) Boxplot summarizing the proportion of CD4^+^PD-1^+^CXCL13^+^ cells from each cell suspension sample, separated according to TGF-β status on HRS cells (determined by IHC). (*E* and *F*) Patient outcomes based on proportion of CD4^+^PD-1^+^CXCL13^+^ T cells in LR-CHL patients. The Kaplan–Meier survival curves are shown for progression-free survival (*E*) and overall survival (*F*). *P* values were calculated using a log rank test.

We hypothesized that cytokines or chemokines produced by HRS cells might influence the TME composition in LR-CHL. Consistent with previous literature (39), we confirmed that CD4^+^PD-1^+^CXCL13^+^ T cells were induced from naïve CD4 T cells by TGF-β in vitro (Fig. 6 *A* and *B*). In addition, IHC analysis revealed that in a subset of LR-CHL patients, HRS cells showed high expression of TGF-β (*n* = 12, 32%) (Fig. 6*C*). Notably, the proportion of CD4^+^PD-1^+^CXCL13^+^ T cells in the region surrounding HRS cells was significantly higher in cases with TGF-β^+^ HRS cells (*P* = 0.02, *t* test) (Fig. 6*D*). These results suggest that TGF-β may play a role in inducing the CD4^+^PD-1^+^CXCL13^+^ T cell population in the LR-CHL TME.

### Outcome Correlation of CD4^+^PD-1^+^CXCL13^+^ T Cells.

We finally investigated the prognostic value of the CD4^+^PD-1^+^CXCL13^+^ T cell population in LR-CHL (*n* = 37) patients uniformly treated with first-line ABVD (doxorubicin, bleomycin, vinblastine and dacarbazine)-like treatment. We observed significantly shortened progression-free survival (PFS) (5-y PFS 71% vs. 92%; *P* = 0.032) and overall survival (OS) (5-y OS 89% vs. 100%; *P* = 0.01) in patients with high levels of CD4^+^PD-1^+^CXCL13^+^ T cells in LR-CHL (Fig. 6 *E* and *F* and *SI Appendix*, Table S5). Importantly, an increased number of PD-1^+^ cells or CXCL13^+^ cells, measured as individual biomarkers, did not correlate with survival (*SI Appendix*, Fig. S9 and Table S6). This difference might reflect a distinct profile of CD4^+^PD-1^+^CXCL13^+^ T cells, as supported by our observations in the single-cell sequencing data, including unique TFH-like characteristics. The other clinical features such as age and advanced stage were not identified as prognostic factors for PFS in univariate analysis (*SI Appendix*, Table S6).

## Discussion

**Fig. 7.**
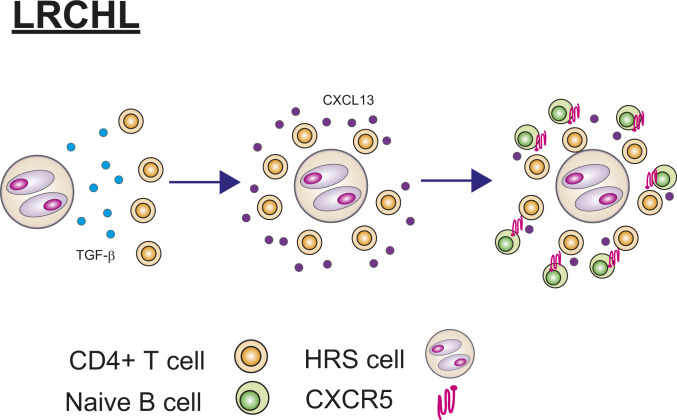
Proposed model of CD4^+^PD-1^+^CXCL13^+^ T cell and HRS cell interactions in LR-CHL. HRS cells secrete TGF-β that induces a CXCL13^+^PD-1^+^ T cell population from CD4^+^ T cells, producing rosettes surrounding the HRS cells. CD4^+^PD-1^+^CXCL13^+^ T cells may in turn attract naïve CXCR5^+^ B cells.

In this study, we comprehensively characterized immune cell populations in the TME of LR-CHL at both the RNA and protein levels. The relative rarity of LR-CHL has hampered its description in the past, and, to the best of our knowledge, this study utilizes one of the largest cohorts to date to investigate the TME of LR-CHL. We identified previously undescribed subpopulations specific to LR-CHL, including CD4^+^CXCL13^+^ T cells that are linked to unique pathological and clinical parameters. CD4^+^CXCL13^+^ T cells form rosettes surrounding HRS cells and coexpress PD-1. To date, PD-1–positive cells in the TME of LR-CHL were considered to be conventional TFH cells (1, 2, 40), but our data demonstrate a distinct phenotype of these CD4^+^PD-1^+^ T cells, implicating the CXCL13/CXCR5 axis. Collectively our results suggest a model in which the microenvironment of LR-CHL is highly organized and in part induced by CD4^+^CXCL13^+^ T cells, which in turn are induced by TGF-β secreted by HRS cells (Fig. 7).

Clinical trials have demonstrated high response rates of up to 87% by PD-1 blockade in relapsed and refractory CHL (17–22), indicating the importance of the PD-1/PD-L1 biology in the disease. Although high efficacy of PD-1 blockade might be associated with high frequency of 9p24.1 alterations in CHL (reported up to 90% response rates) (34–38), previous studies have demonstrated that the proportion of PD-1^+^ T cells is relatively low in CHL (15, 23). In this study, we demonstrated that LR-CHL shows a clearly distinct PD-1/PD-L1 profile when compared to other CHL subtypes, including more PD-1^+^ T cells and fewer *PD-L1* genetic alterations in HRS cells. This is consistent with results from previous small case series in LR-CHL (41, 42). The response of PD-1 blockade by pathological subtype in CHL has not been reported, and further evaluation is warranted. Interestingly, we also observed that coexpression patterns on PD-1^+^CD4^+^ T cells are different among pathological subtypes, and CD4^+^PD-1^+^CXCL13^+^ T cells are specifically enriched in LR-CHL. We also observed a negative correlation between *PD-L1* gene alterations on HRS cells and PD-1 protein expression in the TME of LR-CHL. This supports the notion that PD-L1 expression on HRS cells has a negative impact on PD-1^+^ T cells in LR-CHL, as suggested in a recent publication (43).

CXCL13, which is a ligand of CXCR5, is well known as a B cell attractant via the CXCL13/CXCR5 axis. Consistent with this known feature, CD4^+^ T cells in LR-CHL are located in close proximity to CXCR5^+^ B cells. Moreover, our scRNA-seq data demonstrated enrichment of naïve B cells, indicating that the naïve B cells attracted by CD4^+^CXCL13^+^ T cells might be prevented from entering the germinal center for antigen activation and maturation. The evidence of CD4^+^CXCL13^+^ T cells shaping TME composition may represent a LR-CHL–specific mechanism of immune dysfunction, suggesting that therapeutic targeting of these cells might reverse their immunosuppressive effects (44). Interestingly, therapeutic agents targeting CXCL13/CXCR5 are currently being explored in the context of autoimmune disease and non-Hodgkin lymphoma (40, 45). The characteristics of CD4^+^CXCL13^+^ T cells in LR-CHL are very similar to a CXCL13-producing TFH population that lacks CXCR5 expression identified in breast cancer (44). The scRNA-seq data also demonstrated that CD4^+^CXCL13^+^ T cells have an activated and terminally differentiated phenotype. Consistent with previous reports, CD4^+^PD-1^+^CXCL13^+^ T cells could be induced by TGF-β, a cytokine secreted by HRS cells.

Of clinical importance, our data demonstrated that the presence of CD4^+^PD-1^+^CXCL13^+^ T cells was associated with poor treatment outcome in LR-CHL, suggesting an important role of CD4^+^PD-1^+^CXCL13^+^ T cells in treatment response. In contrast, single IHC positivity of PD-1 and CXCL13 was not associated with outcome, suggesting the importance of identifying specific immune cell subsets using a multiple marker approach. However, it is still unclear whether CD4^+^CXCL13^+^ T cells are the main mediator for chemoresistance to standard chemotherapy, or whether this population is just an ancillary consequence of an HRS cell phenotype that drives poor outcome. In particular, a deeper understanding of receptor/ligand interactions linked to CD4^+^CXCL13^+^ T cells, including the CXCL13/CXCR5 and PD-1/PD-L1 axes, may be beneficial for future therapeutic and biomarker development.

In summary, our results reveal a unique TME composition in LR-CHL. Since the CXCL13/CXCR5 axis could affect multiple types of immune cells, including B cells, FDCs, and T cells, additional investigation into the biology of immune cell interactions will be crucial for future therapeutic development of alternative checkpoint inhibitors.

## Materials and Methods

Additional detailed materials and methods are available in *SI Appendix*, *Materials and Methods*.

### Tissue Samples.

For single-cell RNA sequencing, a total of 28 patients with histologically confirmed CHL (8 LR, 11 NS, 9 MC) and 5 patients with reactive lymphoid hyperplasia (but no evidence of malignant disease or systemic autoimmune disease) were included in this study. Patients were selected based on the availability of tissue that had been mechanically dissociated and cryopreserved as cell suspensions following diagnostic lymph node procedures at British Columbia (BC) Cancer.

The independent validation cohort of LR-CHL patients consisted of 31 newly diagnosed cases at BC Cancer between 2000 and 2018. The median follow-up time for living LR-CHL patients was 7 y (range: 1.2 to 17.4 y). Patient characteristics are summarized in *SI Appendix*, Tables S1, S2, S4, and S5.

This study was reviewed and approved by the University of British Columbia-BC Cancer Agency Research Ethics Board (H14-02304), in accordance with the Declaration of Helsinki. We obtained written informed consent from the patients or informed consent was waived for the samples used in this retrospective study.

### Single-Cell RNA Sequencing and Library Preparation.

Samples were processed and libraries were prepared for scRNA-seq as previously described (15). In brief, sorted cells from cell suspensions were collected, and 8,700 cells per sample were loaded into a Chromium Single Cell 3′ Chip Kit v2 (PN-120236). Libraries were constructed using the Single Cell 3′ Library and Gel Bead Kit v2 (PN-120237) and Chromium i7 Multiplex Kit (PN-120262). For further details, see *SI Appendix*, *Materials and Methods*.

### Normalization and Batch Correction.

Normalization and batch correction were performed as previously described (15). Briefly, CellRanger count data from all cells (*n* = 150,611) was read into R (v3.6.1) to create a single “SingleCellExperiment” object. To remove batch effects resulting from different library preparation chips, the fast mutual nearest neighbors (MNN) batch correction technique in the scran package (46) (v1.14.5) was utilized, grouping cells by their chip and using the expression of genes with positive biological components. For further details, see *SI Appendix*, *Materials and Methods*.

### Clustering and Annotation.

Unsupervised clustering was performed with the PhenoGraph algorithm (47) as previously described (15). For visualization purposes, t-distributed stochastic neighbor embedding transformation was performed using the first 10 MNN-corrected components as input. For further details, see *SI Appendix*, *Materials and Methods*.

### MC-IF on TMA, Scanning, and Image Analysis.

MC-IF was performed as previously described (15). In brief, TMA slides were deparaffinized and incubated with antibodies to each marker of interest (CXCR5, CXCL13, BCL6, CD20, PD-1, CD4, and CD30), followed by detection using Mach2 horseradish peroxidase and visualization using Opal fluorophores (*SI Appendix*, Table S7). To analyze the spectra for all fluorophores included, inForm image analysis software (v2.4.10; PerkinElmer) was used. For further details, see *SI Appendix*, *Materials and Methods*.

### Cell Isolation and Purification of Human T Cells.

We purified CD4^+^ T lymphocytes from peripheral blood mononuclear cells (PBMCs) (see *SI Appendix, Materials and Methods* for details). Isolated naïve CD4^+^ cells were incubated in culture medium with or without TGF-β. At the end of day 5, we washed and analyzed the T cells using flow cytometry for characterization.

### Flow Cytometry.

To characterize T cells in vitro, we stained cells with a panel of antibodies, including CD3, CD4, PD-1, and CXCL13 (see *SI Appendix*, *Materials and Methods* and Table S8 for details), and assessed them using flow cytometry (FACSymphony, BD). Flow cytometry data were analyzed using FlowJo software (v10.2; TreeStar).

### Survival Analysis.

OS (death from any cause) and PFS (the time from initial diagnosis to the date of disease progression or relapse/death from any cause) were analyzed using the Kaplan–Meier method, and results were compared using a log rank test. Survival analyses were performed in the R Statistical Environment (v3.6.1). For further details, see *SI Appendix*, *Materials and Methods*.

## Supplementary Material

Supplementary File

Supplementary File

## Data Availability

Single cell RNA-seq counts (generated with CellRanger v2.1.0) and a merged ‘SingleCellExperiment’ R object is available in the European Genome-phenome Archive (EGA) (EGAS00001005541) via controlled access.
