# Supplementary material for: Single-cell profiling reveals the importance of CXCL13/CXCR5 axis biology in lymphocyte-rich classic Hodgkin lymphoma
Source: Proc Natl Acad Sci U S A. 2021 Oct 6;118(41):e2105822118. doi: 10.1073/pnas.2105822118 (PMC8521678; doi:10.1073/pnas.2105822118)
Supplement: Supplementary File [file pnas.2105822118.sapp.pdf]

PNAS
www.pnas.org

SUPPLEMENTAL DATA

Single cell profiling reveals the importance of CXCL13/CXCR5 axis biology in lymphocyte rich classic Hodgkin lymphoma

Tomohiro Aoki[1,2*], Lauren C. Chong[1*], Katsuyoshi Takata[1,3], Katy Milne[4,5], Ashley Marshall[4,5], Elizabeth A. Chavez[1], Tomoko Miyata-Takata[1], Susana Ben-Neriah[1], Doria Unrau[4,5], Adele Telenius[1], Merrill Boyle[1], Andrew P. Weng[6], Kerry J. Savage[1], David W. Scott[1], Pedro Farinha[1,2], Sohrab P. Shah[2,7,8], Brad H. Nelson[4,9] and Christian Steidl[1]

[1]Centre for Lymphoid Cancer, BC Cancer, Vancouver, British Columbia, Canada.
[2]Department of Pathology and Laboratory Medicine, University of British Columbia, Vancouver, British Columbia, Canada.
[3]Division of Molecular and Cellular Pathology, Niigata University Graduate School of Medical and Dental Sciences
[4]Deeley Research Centre, BC Cancer, Victoria, British Columbia, Canada.
[5]Department of Biochemistry and Microbiology, University of Victoria, Victoria, British Columbia, Canada.
[6]Terry Fox Laboratory, BC Cancer, Vancouver, British Columbia, Canada.
[7]Department of Molecular Oncology, BC Cancer, Vancouver, British Columbia, Canada.
[8]Department of Epidemiology and Biostatistics, Memorial Sloan Kettering Cancer Center, New York, New York.
[9]Department of Medical Genetics, University of British Columbia, Vancouver, British Columbia, Canada
[*] - equal contribution

Corresponding Author:

Christian Steidl, MD

BC Cancer Research Centre,

675 West 10th Ave, Room 12-110, Vancouver, BC V5Z 1L3, Canada

E-mail: CSteidl@bccancer.bc.ca

Phone (office): 604-675-8046
FAX:604-675-8183

This PDF file includes:

Supplementary text
Figures S1 to S10
SI References
Tables S1 to S8

Other supplementary materials for this manuscript include the following:

Dataset 1

Supplementary Information Text

Materials and Methods

Single cell RNA sequencing sample preparation. Samples for scRNA-seq were prepared as previously described[1]. In brief, cell suspensions from CHL tumors or reactive lymph node were rapidly thawed at 37°C, washed in 10ml of RPMI1640/10% fetal bovine serum (FBS) solution DNase I (Millipore Sigma, Darmstadt, Germany) and washed in PBS. Cells were resuspended in PBS containing 3% FBS and stained with DAPI (Sigma-Aldrich, St. Louis, MO) for 15 min at 4°C in the dark. Viable cells were sorted on a FACS Fusion (BD Biosciences) using an 85 µm nozzle. Sorted cells were collected in 0.5 ml of medium, centrifuged and diluted in RPMI1640/10% FBS.

Library Preparation and single-cell RNA sequencing. In total, 8700 cells per sample were loaded into a Chromium Single Cell 3' Chip kit v2 (PN-120236) and processed according to the Chromium Single Cell 3' Reagent kit v2 User Guide. Libraries were constructed using the Single Cell 3' Library and Gel Bead Kit v2 (PN-120237) and Chromium i7 Multiplex Kit (PN-120262). Single cell libraries from two samples were pooled and sequenced on one HiSeq 2500 125 base PET lane. CellRanger software (v2.1.0; 10X Genomics) was used to demultiplex the raw data, generate quality metrics, and generate per-gene count data for each cell.

Normalization and batch correction. Analysis and visualization of scRNA-seq data was performed as described previously[1] in the R statistical environment (v3.6.1).

CellRanger count data from all cells (n = 150,611) were read into a single 'SingleCellExperiment' object. Cells were filtered if they had ≥ 20% reads aligning to mitochondrial genes, or if their total number of detected genes was ≥ 3 median absolute deviations from the sample median. This yielded a total of 146,437 cells for analysis. The scran package (v1.14.5) was used to compute cell-specific sum factors with the method described by Lun et al[2]. Briefly, quick-clustering was performed to identify pools of cells with similar expression profiles, and size factors were calculated for each pool by normalizing summed expression profiles against the full set of expression profiles. The pool-based size factors were then deconvolved to generate sum factors for each cell[2]. The scater package (v1.14.6) was used to log-normalize the count data using the cell-specific sum factors.

To remove batch effects resulting from different chips and library preparation, the mutual nearest neighbors (MNN) batch correction technique in the scran package was utilized. Biologically relevant genes were first identified by fitting a mean-dependent trend to the gene-specific variances, then decomposing the variance into biological and technical components and selecting genes with positive biological components. Fast MNN correction was then performed on the expression of these genes, grouping cells by their chip. This produced a matrix of corrected low-dimensional (d = 50) coordinates for each cell, which was used as input for downstream analyses.

Clustering and annotation. Unsupervised clustering was performed with the PhenoGraph algorithm[4], using the first 10 MNN-corrected components as input. Clusters from PhenoGraph were manually assigned to a cell type by comparing the mean

expression of known markers across cells in a cluster. Markers used to type cells included CD19 (B cells), CD27, IGHD (Naïve B cell), CD8, CD3, CD4 (T cells), NCAM1 (NK cells), GZMA, GZMK (cytotoxic T cells, NK cells), FOXP3, IL2RA, IKZF2, CTLA4, LAG3, TNFRSF18 (Tregs), IFNG, TBX21 (Th1 cells) GATA3, IL4, IL13 (Th2 cells), CD161, CCR4 (Th17 cells), PDCD1, CXCR5, BCL6 (TFH cells), CCR7, IL7R, LEF1 (Naive T cells), CD25, CD69 (activated T cells) and CD68 (Macrophages). $CD4^+$ helper T cells (as defined by cluster assignment) were further classified into subsets according to positivity (normalized log-transformed expression > 0) and negativity of various markers. The following criteria were used: (1) Th1 cells were positive for either IFNG or TBX21 (T-bet), and negative for CCR4; (2) Th2 cells were positive for any of IL4, IL13, PTGDR2 (CD294), or GATA3; (3) TFH cells were positive for at least 2 of PDCD1 (PD-1), CXCR5 and BCL6, and negative for both CCR7 and FOXP3; (4) Th17 cells were positive for both KLRB1 (CD161) and CCR4. We also used marker expression to identify TFH-specific subsets using the following criteria: (1) “CXCL13+CXCR5- TFH” cells were positive for PDCD1 (PD-1), CXCL13 and ICOS, and negative for CXCR5; (2) “CXCL13-CXCR5+ TFH” cells were positive for either PDCD1 (PD-1) or ICOS, positive for CXCR5, and negative for CXCL13.

For visualization purposes, tSNE transformation was performed with the scater package using the first 10 MNN-corrected components as input. All differential expression results were generated using the *findMarkers* function of the scran package, which performs gene-wise t-tests between pairs of clusters, and adjusts for multiple testing with the Benjamini-Hochberg method.

Diffusion map analysis. To examine cell trajectories in the scRNA-seq data, the diffusion map algorithm[5] was run as implemented in the scater package using the first 10 MNN-corrected components as input. To identify potential signatures associated with the first two diffusion map dimensions, gene signature lists described in Azizi et al. were used[3]. For each signature, the Pearson correlation between expression of each gene and the dimension score was calculated, and the mean correlation value across all genes in the signature was taken (***SI Appendix*, Fig. S6C-D**). Additionally, individual genes most highly related to the first two dimensions were identified by calculating Pearson correlations for every gene, and expression of the top 4 positively/negatively correlated genes were visualized in diffusion map space (**Fig. 2G, *SI Appendix*, Fig. S6B**).

iTALK analysis. The iTALK R toolkit[6] (v0.1.0) was used to identify potential receptor/ligand interactions in the single-cell RNAseq data. Cells were classified into broad subtypes based on their cluster assignments (i.e. T helper cells, B cells), and for each subtype differential expression (DE) was performed between cells originating from LR-CHL samples and other CHL samples (see above for DE methodology). The DE results labeled by cell type were passed into the *FindLR* function (datatype = "DEG") to identify interactions for all communication types. Interactions were ordered using a score calculated by multiplying the log fold-change of both cell types (cell_from_logFC * cell_to_logFC) and taking the absolute value. The top 10 interactions with the highest score were passed to the *LRPlot* function for visualization.

Tissue microarray (TMA) construction, single color IHC. For TMA construction, 1.5mm duplicate cores were obtained from representative areas containing HRS cells of 37 diagnostic biopsies of LR-CHL. The diagnosis was made according to the WHO classification[7]. For IHC staining, 4μm slides of the TMA and antibodies listed in ***SI Appendix*, Table S6** were used. Staining was performed on a Benchmark XT platform (Roche Diagnostics, USA) or intelliPATH platform (Biocare Medical, USA). The slides were independently scored by KT, TT and PF. Evaluation of tumor and microenvironment cells (CD20, CXCL13, CXCR5, CD3, TGF-β, PD-1 and PD-L1) was performed, and relative percentage of positive cells in relation to overall cellularity (scored from 0-100% in 10% increments) was reported as an average of both duplicate cores as previously described[8]. PD-L1 and TGF-β expression were quantified by assigning a histoscore out of 300, which was calculated by multiplying the value for staining intensity (0-3) with the percentage of positive cells (0-100%). An optimal cut-off for the histoscore was chosen by a hematopathologist to produce the strongest discrimination between the respective groups. PD-L1 protein expression positivity (on HRS cells) was defined as ≥ 80% positive HRS cells. TGF-β protein expression positivity (on HRS cells) was defined as ≥ 10% positive HRS cells. The thresholds used to assign positivity for other IHC markers were determined by selecting the optimal values to maximize Cox proportional hazard ratios on PFS and OS. This analysis was performed in R (4.0.2) using the survMisk package (0.5.5). To assess the percentage of HRS cells surrounded by $CXCL13^+$ and $PD\text{-}1^+$ T cells, we counted the number of tumor cells in each core and determined the percentage of HRS cells with a rosetting T cell pattern. A

rosetted HRS cell was defined as one in which greater than 50% of the cell's circumference was occupied by $CXCL13^+$ or $PD\text{-}1^+$ cells[9].

Multi-color IHC on TMA, scanning and image analysis. TMA slides were deparaffinized in xylene and rinsed with dH_2O. Antigen retrieval was performed in AR6 buffer (PerkinElmer, USA) with Diva decloaker (Biocare Medical, USA). The primary antibody for PD-1 was incubated for 30min in an Intellipath FLX rack at room temperature, followed by detection using the Mach2 mouse HRP with 10 min incubation. Visualization of PD-1 was achieved using Opal 620. The slide was placed into AR6 buffer and heated using a microwave. In serial order, the slide was incubated with primary antibody for CD4, followed by detection using Mach2 mouse HRP, and visualization was accomplished using Opal 690. The slide was again placed into AR6 buffer and heated using a microwave. Then the primary antibody for BCL6 was incubated, followed by detection of Mach2 mouse HRP and Opal 520 for visualization. The slide was placed into AR6 buffer for microwaving. The primary antibody for CD20 was incubated, followed by detection of Mach2 rabbit HRP and visualization for Opal 540. Microwave heating was repeated with AR6 buffer. The primary antibody for CXCR5 was incubated, followed by detection of Mach2 rabbit HRP and visualization for Opal 570. Microwave heating was repeated again with AR6 buffer. The primary antibody for CD30 was incubated, followed by detection of Mach2 HRP and visualization for Opal 650. The primary antibody for CXCL13 was incubated, followed by detection of Mach2 HRP and visualization for Opal 480. Nuclei were visualized with DAPI staining and the section was coverslipped using Fluoro Care Anti-Fade Mountant. TMA slides were

scanned using the Vectra multispectral imaging system (v3.0.7; PerkinElmer, USA) following manufacturer's instructions to generate .im3 image cubes for downstream analysis. Optimal exposure times for fluorophores ranged between 50 and 200ms. To analyze the spectra for all fluorophores included, inForm image analysis software (v2.4.10; PerkinElmer, USA) was used. Cells were first classified into tissue categories using DAPI and CD30 to identify $CD30^{+}DAPI^{+}$, $CD30^{-}DAPI^{+}$, and $CD30^{-}DAPI^{-}$ areas via manual circling and training. The $CD30^{+}DAPI^{+}$ regions were considered to be HRS-surrounding regions. Cells were then phenotyped as positive or negative for each of the seven markers (PD-1, CXCR5, CXCL13, BCL6, CD20, CD4 and CD30) using a supervised machine learning approach trained on manually annotated positive/negative cells in representative images (inForm method). Data were merged in R by X-Y coordinates so that each cell could be assessed for all markers simultaneously. Nearest neighbor analysis was performed with the spatstat R package (v1.64-1). Within-distance and density analyses were performed using the rtree package (v0.1.0).

Cell isolation and purification of human T cells. We purified $CD4^{+}$ T lymphocytes from peripheral blood mononuclear cells (PBMCs) by negative selection using the untouched CD4 T cell Isolation Kit II (Miltenyi Biotec, Auburn, CA) according to the manufacturer's instructions. Isolated $CD4^{+}$ cells were stimulated with soluble monoclonal antibodies to CD3 (2 μg/ml, OKT3, eBioscience, Sandiego, CA, USA) and CD28 (1 μg/ml, clone CD28.2, Biolegend, Sandiego, CA, USA) and incubated in culture medium in the presence or absence of TGF-β (10 ng/ml, Cell Signaling Technology, Danvers, MA, USA) in Iscove's Modified Dulbecco's Media (IMDM) GlutaMAX™ supplement (Life

Technologies) supplemented with 10% fetal bovine serum (FBS) solution, and 100 U/ml penicillin-streptomycin (Thermo Fisher Scientific, Waltham, MA, USA). At the end of day 5, we washed and analyzed the T cells using flow cytometry for characterization.

Flow cytometry. To characterize T cells *in vitro*, we used a panel of antibodies (described in ***SI Appendix*, Table S7**) and Live/Dead Fixable Yellow (Life Technologies). Briefly, cells from T cell cultures were washed in PBS/2% FBS solution. Cell suspensions from CHL tumors or reactive lymph node were rapidly defrosted at 37C, washed in 10ml of RPMI1640/10% fetal bovine serum (FBS) solution containing DNase I (Millipore Sigma, Darmstadt, Germany) and washed in PBS. Cells were stained with the antibody cocktail for 15 minutes on ice in the dark and assessed using flow cytometry (LSRFortessa or FACSymphony, BD, Franklin Lakes, NJ, USA). For cytokine intracellular staining, cells were permeabilized using the Fixation/Permeabilization Solution Kit (BD) according to the manufacturer's instructions. Flow cytometry data were analyzed using FlowJo software (v10.2; TreeStar, Ashland, OR, USA) (***SI Appendix*, Fig. S10**). Statistical analyses were performed using GraphPad Prism Version 7 (GraphPad Software Inc., La Jolla, CA).

Fluorescence immunophenotyping and interphase cytogenetics as a tool for investigation of neoplasms. Fluorescence immunophenotyping and interphase cytogenetics as a tool for investigation of neoplasms (FICTION) was performed as previously described.[10,11]. In brief, formalin-fixed paraffin-embedded tissue (FFPET) tumors, represented as duplicate 1.5 mm cores on tissue microarray (TMA) were

subjected to immunohistochemistry for CD30 (Primary antibody: Monoclonal mouse anti-human CD30, clone Ber-H2, DAKO; Secondary antibody: Alexa-Fluor 594 goat anti-mouse IgG, Invitrogen). Then, probe and target DNA were co-denatured for 5 min at 83C and hybridized at 37C overnight. The following in-house bacterial artificial chromosome (BAC) clones targeting 9p24.1 chromosome was used: RP11-963L3 and RP11-12D24 (PD-L1), RP11-207C16 and RP11-845C2 (PD-L2) as previously described[11]. The frequency of false positive amplifications for the BAC FISH probe was established by hybridization to reactive lymph node cells with mean proportion of cells with 3 or more signals of 7.5%. For the purpose of this study the cut-off value for true gains (3-5 signals) was set at > 20% and the cut-off value for amplifications (6 or more signals) was set at 10%, scoring a minimum of 20 interphase $CD30^+$ cells. Slides were analyzed using a Carl Zeiss Axio lmager Z2 microscope equipped with a Plan Apochromat 63x/1.4 oil objective. The images were acquired using a Cool Cube Digital CCD and Metasystems software (version 5.5.10).

Survival analysis. Overall survival (OS, death from any cause) and progression-free survival (PFS, the time from initial diagnosis to the date of disease progression or relapse/death from any cause) were analyzed using the Kaplan-Meier method and results were compared using a log rank test. Univariate Cox regression analyses were performed to assess the effects of prognostic factors. High proportion of PD-1 positive cells was defined as ≥ 10% positive leukocytes as described before[12]. The cutoffs for proportions of $CXCL13^+$ cells were determined by Cox proportional hazard based analyses using survMisk package. High proportion of CXCL13 positive cells was defined as greater than

5% positive leukocytes. The cutoffs for proportions of $CD4^+PD\text{-}1^+CXCL13^+$ cells was determined by Cox proportional hazard based analyses using survMisk package. High proportion of $CD4^+PD\text{-}1^+CXCL13^+$ cells was defined as greater than 2% positive cells in MC-IHC data. Survival analyses were performed in the R statistical environment (v3.6.1).

Statistical results & visualization. All t-tests reported are two-sided Student's t-tests, and P-values < 0.05 were considered to be statistically significant. Fisher's exact test was used to compare clinical and patient characteristics. In all box plots, boxes represent the interquartile range with a horizontal line indicating the median value. Whiskers extend to the farthest data point within a maximum of 1.5 × the interquartile range, and colored dots beyond the whiskers represent outliers.

Data availability. Single cell RNA-seq counts (generated with CellRanger v2.1.0) and a merged 'SingleCellExperiment' R object will be available in the EGA (EGAS00001005541) via controlled access

SI References

1. Aoki T, Chong LC, Takata K, et al. Single-Cell Transcriptome Analysis Reveals Disease-Defining T-cell Subsets in the Tumor Microenvironment of Classic Hodgkin Lymphoma. *Cancer Discov.* 2020;10(3):406-421.
2. Lun AT, Bach K, Marioni JC. Pooling across cells to normalize single-cell RNA sequencing data with many zero counts. *Genome Biol.* 2016;17:75.
3. Azizi E, Carr AJ, Plitas G, et al. Single-Cell Map of Diverse Immune Phenotypes in the Breast Tumor Microenvironment. *Cell.* 2018;174(5):1293-1308 e1236.
4. Levine JH, Simonds EF, Bendall SC, et al. Data-Driven Phenotypic Dissection of AML Reveals Progenitor-like Cells that Correlate with Prognosis. *Cell.* 2015;162(1):184-197.
5. Haghverdi L, Buettner F, Theis FJ. Diffusion maps for high-dimensional single-cell analysis of differentiation data. *Bioinformatics.* 2015;31(18):2989-2998.
6. Wang Y, Wang R, Zhang S, et al. iTALK: an R Package to Characterize and Illustrate Intercellular Communication. *BioRxiv.* 2019.
7. SH S, E C, NL H, et al. WHO Classification of Tumours of Haematopoietic and Lymphoid Tissues. Revised 4th ed. Lyon, France: International Agency for Research on Cancer (IARC). 2017.
8. Steidl C, Lee T, Shah SP, et al. Tumor-associated macrophages and survival in classic Hodgkin's lymphoma. *N Engl J Med.* 2010;362(10):875-885.
9. Falini B, Bigerna B, Pasqualucci L, et al. Distinctive expression pattern of the BCL-6 protein in nodular lymphocyte predominance Hodgkin's disease. *Blood.* 1996;87(2):465-471.
10. Steidl C, Shah SP, Woolcock BW, et al. MHC class II transactivator CIITA is a recurrent gene fusion partner in lymphoid cancers. *Nature.* 2011;471(7338):377-381.
11. Twa DD, Chan FC, Ben-Neriah S, et al. Genomic rearrangements involving programmed death ligands are recurrent in primary mediastinal large B-cell lymphoma. *Blood.* 2014;123(13):2062-2065.
12. Hollander P, Kamper P, Smedby KE, et al. High proportions of PD-1(+) and PD-L1(+) leukocytes in classical Hodgkin lymphoma microenvironment are associated with inferior outcome. *Blood Adv.* 2017;1(18):1427-1439.

Supplementary Figures

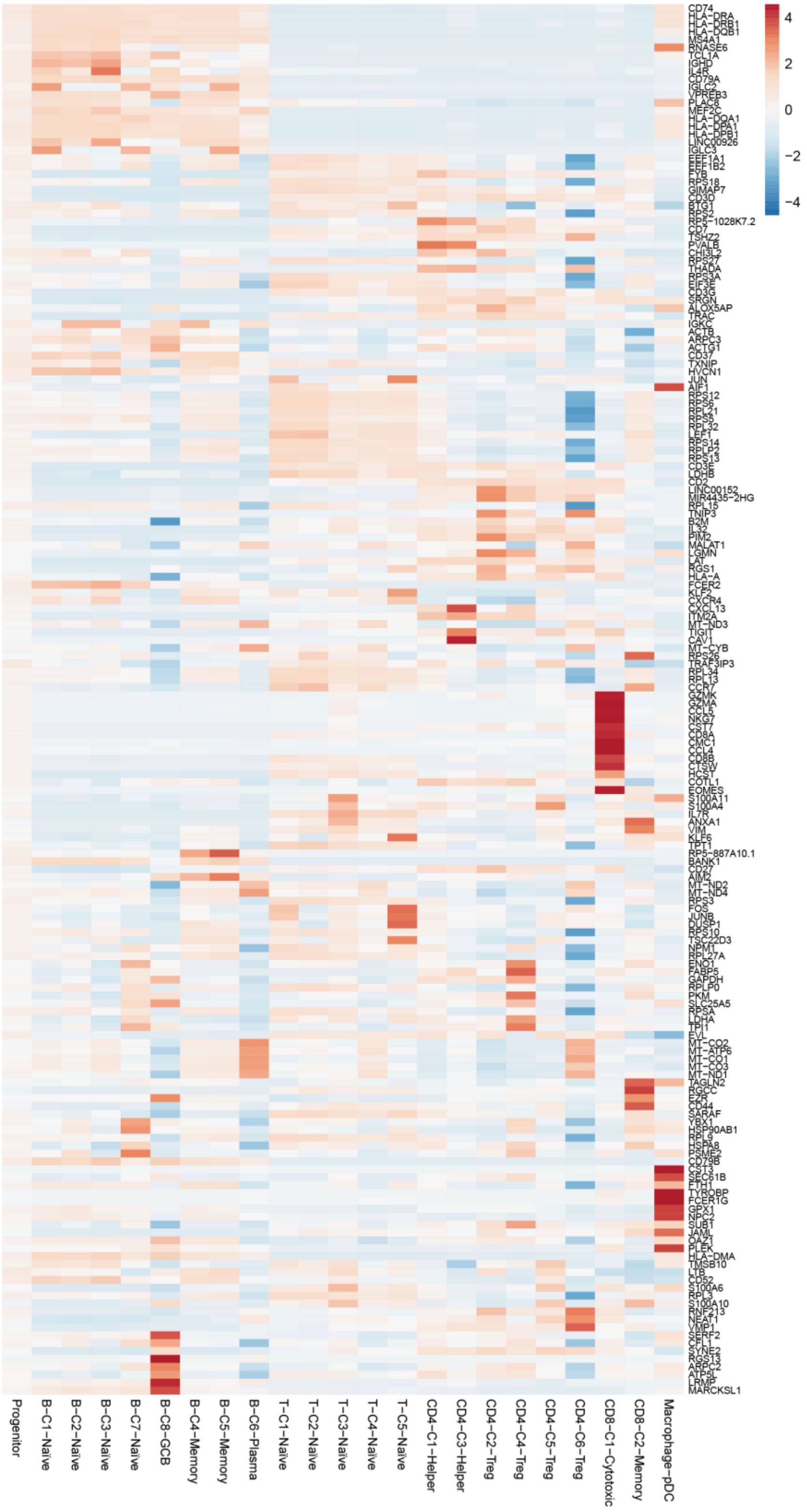

Supplemental Figure 1

Fig. S1. Expression of the top discriminatory genes in each cluster. Differential

expression was performed according to cluster (see ***SI Appendix, Materials and Methods***). Mean expression of the top 20 discriminatory genes for each cluster is shown (data has been scaled row-wise).

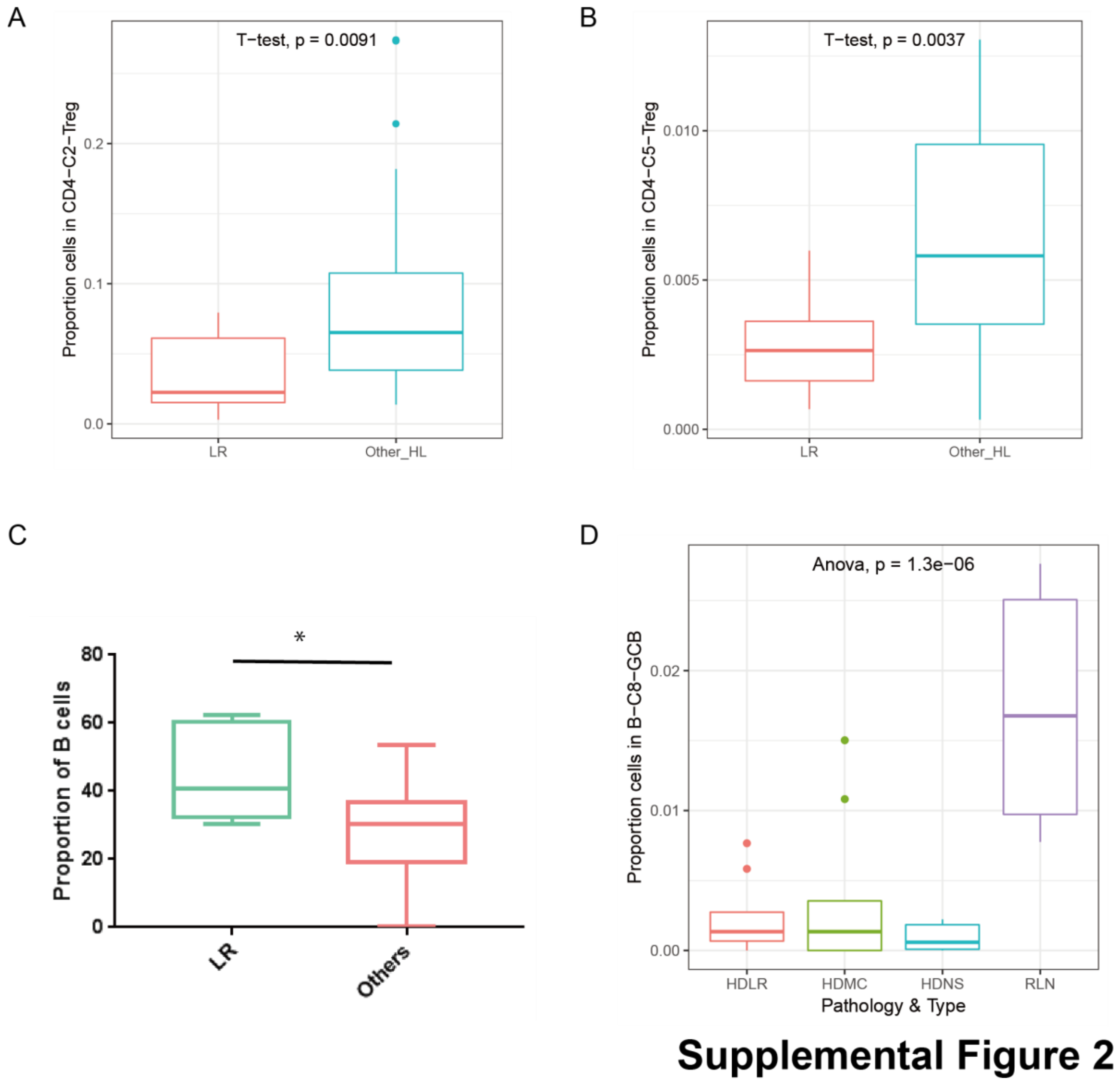

Fig. S2. Proportion of cells assigned to immune cell subsets. (*A*-*B*) Box plots summarizing the proportion of type 1 Treg (*A*; cluster "CD4-C2-Treg") and classical FOXP3^{+} Treg (*B*; cluster "CD4-C5-Treg") cluster cells in each sample, separated

according to CHL subtype. (*C*) Box plot showing the proportion of B cells identified by flow cytometry for all samples, grouped by pathological subtype (n = 6). Data are shown as the mean ± SEM (*P < 0.05). (*D*) Box plot showing the proportion of germinal center B cells (cluster "B-C8-GCB") for all samples, grouped by pathological subtype.

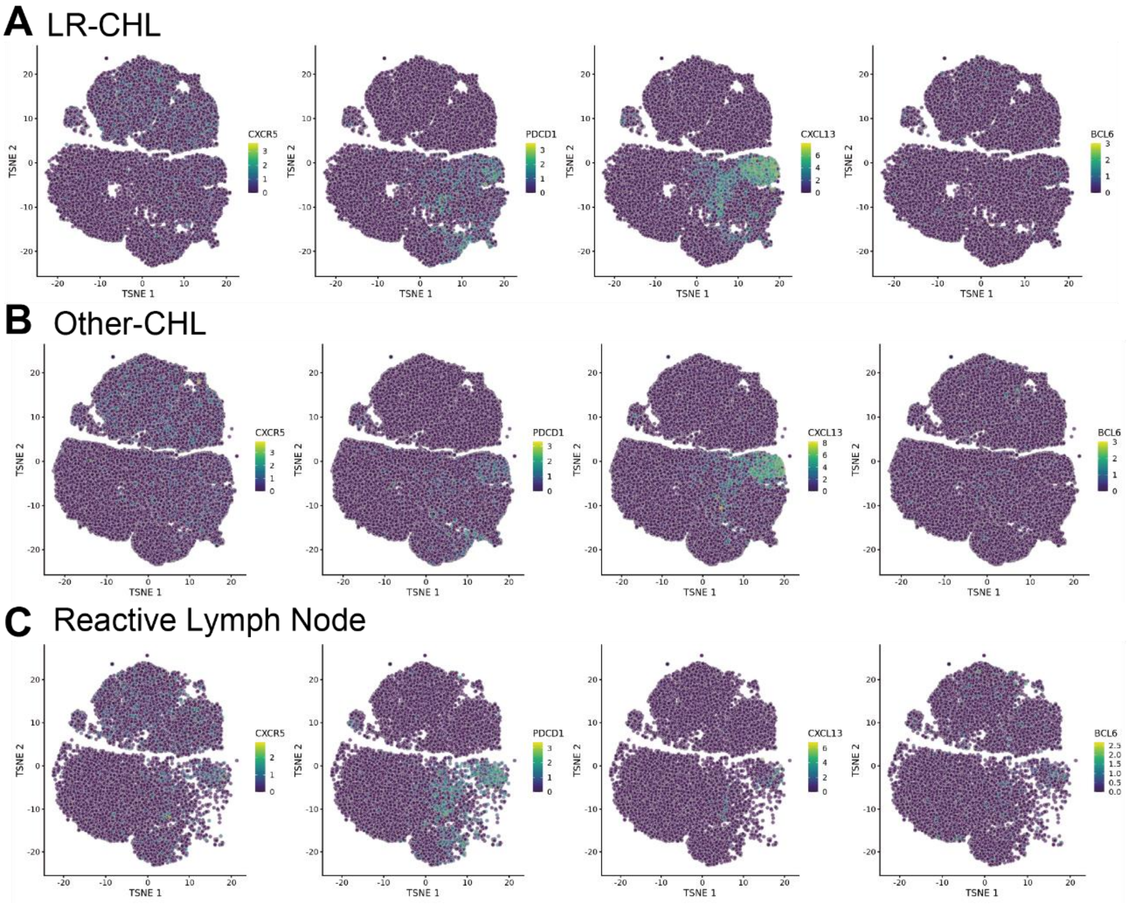

Supplemental Figure 3

Fig. S3. Expression of canonical markers for a follicular helper T cell phenotype (CXCR5, PDCD1 (PD-1), CXCL13 and BCL6) is shown for all cells in tSNE space according to disease subtype.

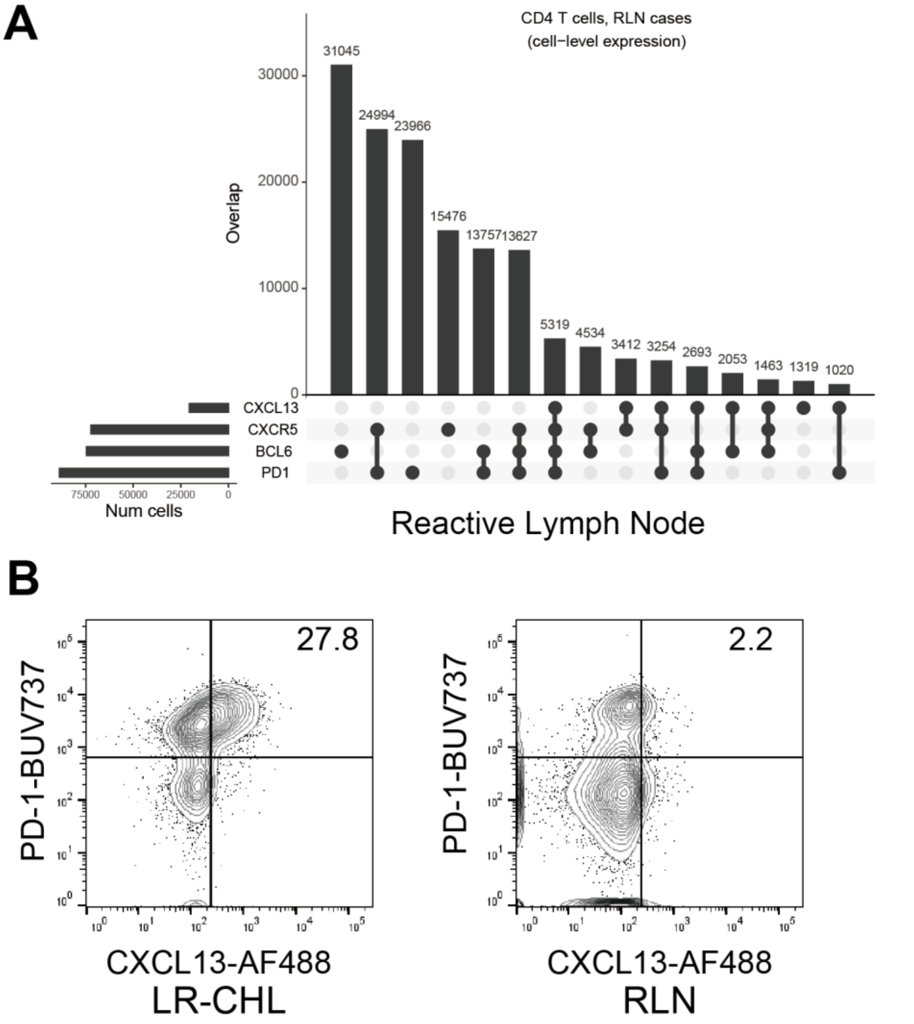

Supplemental Figure 4

Fig. S4. Co-expression pattern of TFH markers measured by flow cytometry. (*A*) UpSet plot showing co-expression patterns on CD4+ T cells in RLN by flow cytometry. (*B*) Representative flow cytometric analysis of PD-1 and CXCL13 expression on CD4+ T cells from a primary LR-CHL patient sample (left) or RLN (right).

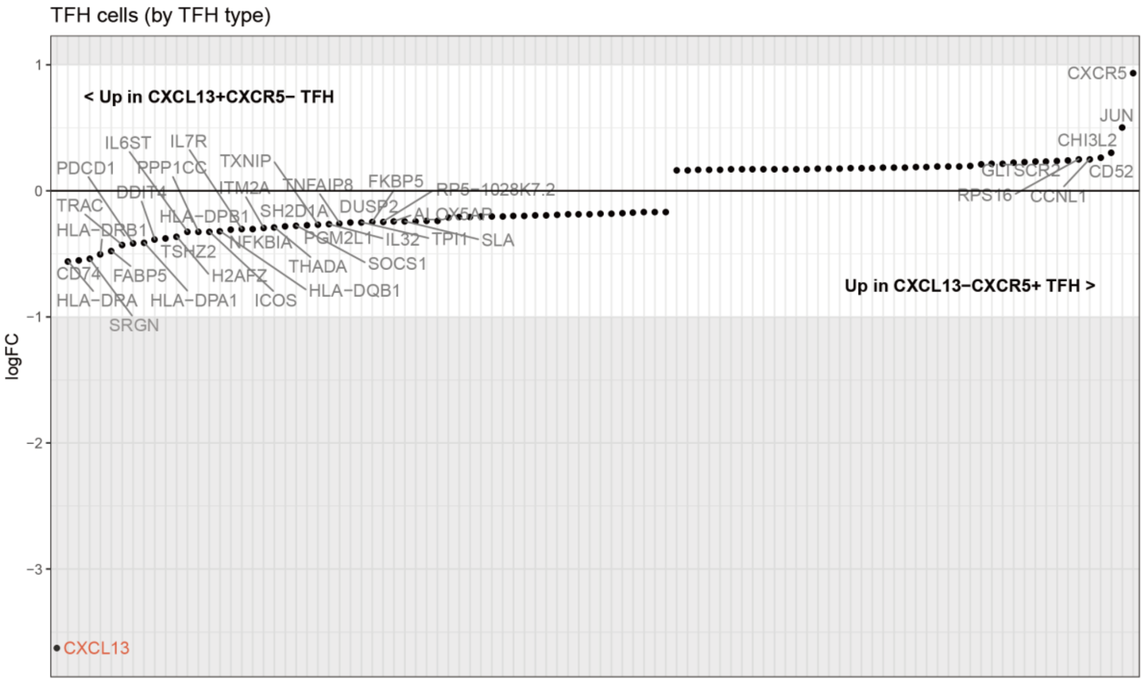

Supplemental Figure 5

Fig. S5. Differentially expressed genes between CXCL13^{+}CXCR5^{-} and CXCR5^{+}CXCL13^{-} cells in the helper T cell clusters. The top 100 genes by absolute log2 fold-change are shown on the x-axis, ordered by ascending log fold-change on the y-axis. The top 40 genes are labeled.

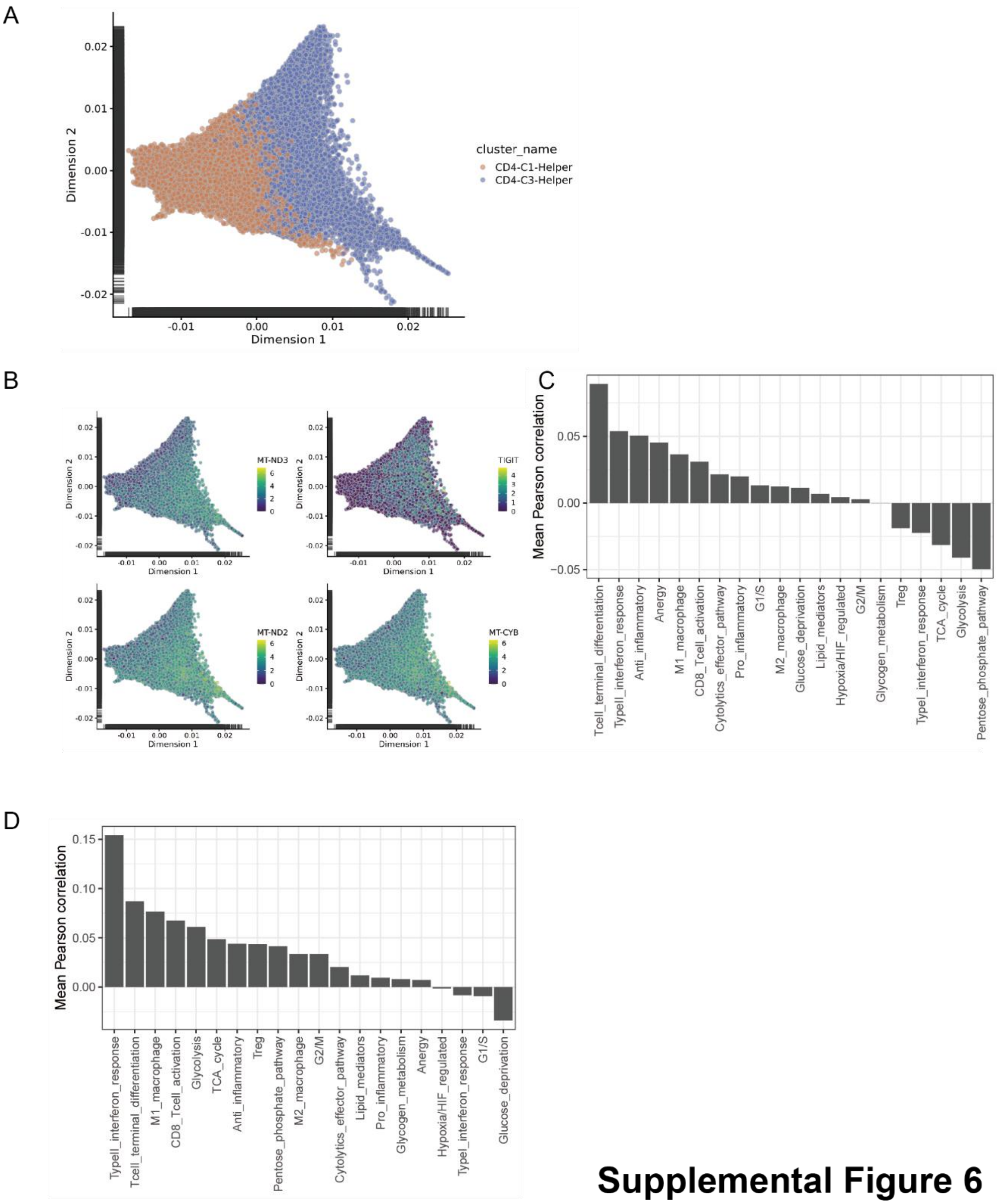

Supplemental Figure 6

Fig. S6. Association of diffusion map dimensions with cell signatures and individual genes. (*A*) Cellular trajectories were inferred using diffusion map analysis of cells in all CD4$^+$ helper T cell clusters (“CD4-C1-Helper” and “CD4-C3-Helper”). Individual cells are shown in the first two resulting dimensions, and are colored according to cluster

assignment. (*B*) The expression of the 4 genes that were most positively correlated with dimension 1 score is shown in diffusion map space. (*C-D*) Bar graphs showing mean Pearson correlation between the genes in each signature (see ***SI Appendix, Materials and Methods***) and diffusion map scores for dimension 1 (*C*) and 2 (*D*).

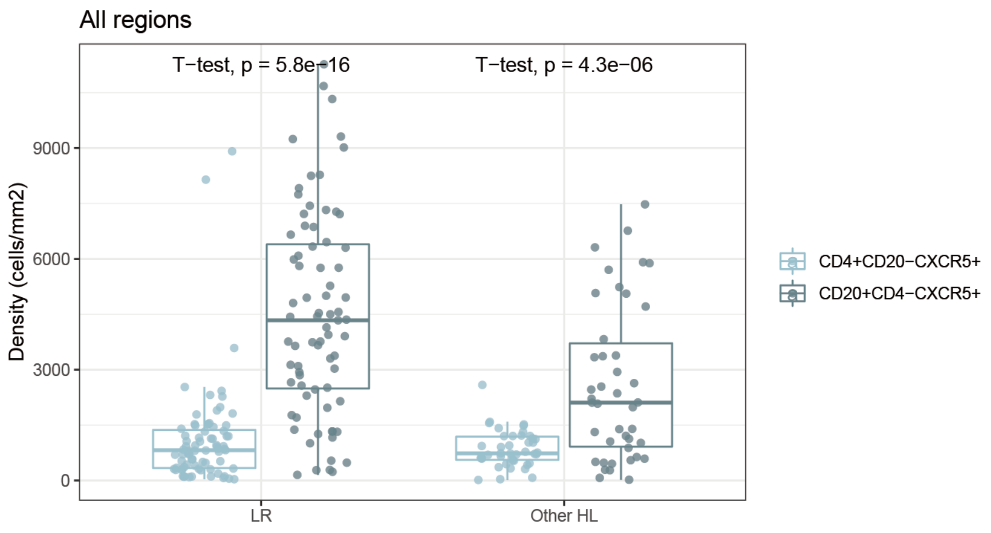

Supplemental Figure 7

Fig. S7. Density of CXCR5^{+} cells in LR and other CHL. Box plot showing the density of CD4^{+}CD20^{-}CXCR5^{+} T cells and CD20^{+}CD4^{-}CXCR5^{+} B cells in each histological subtype as assessed by multicolor IHC.

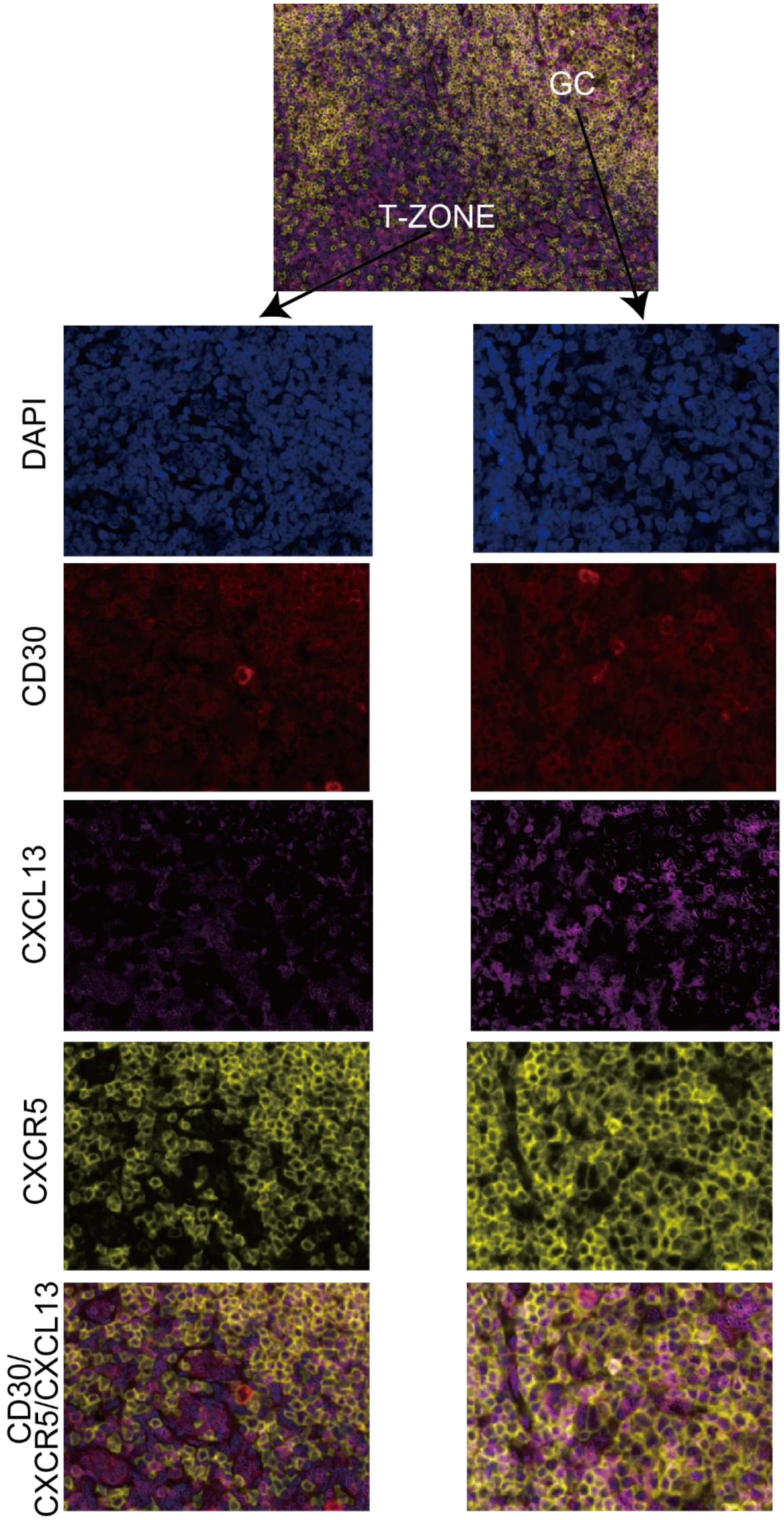

Supplemental Figure 8

Fig. S8. Localization of CXCL13/CXCR5 positive cells in RLN tissue. Multicolor IF staining in RLN for CD30 (red), CXCL13 (magenta) and CXCR5 (yellow) shows localization of CXCR5^{+} cells near CXCL13^{+} cells in the region of a germinal center (GC) and a T cell rich zone (T-ZONE) in reactive lymph node tissue. Cells with a typical TFH cell phenotype (PD1^{+}CXCR5^{+}CXCL13^{-}) are enriched in these areas.

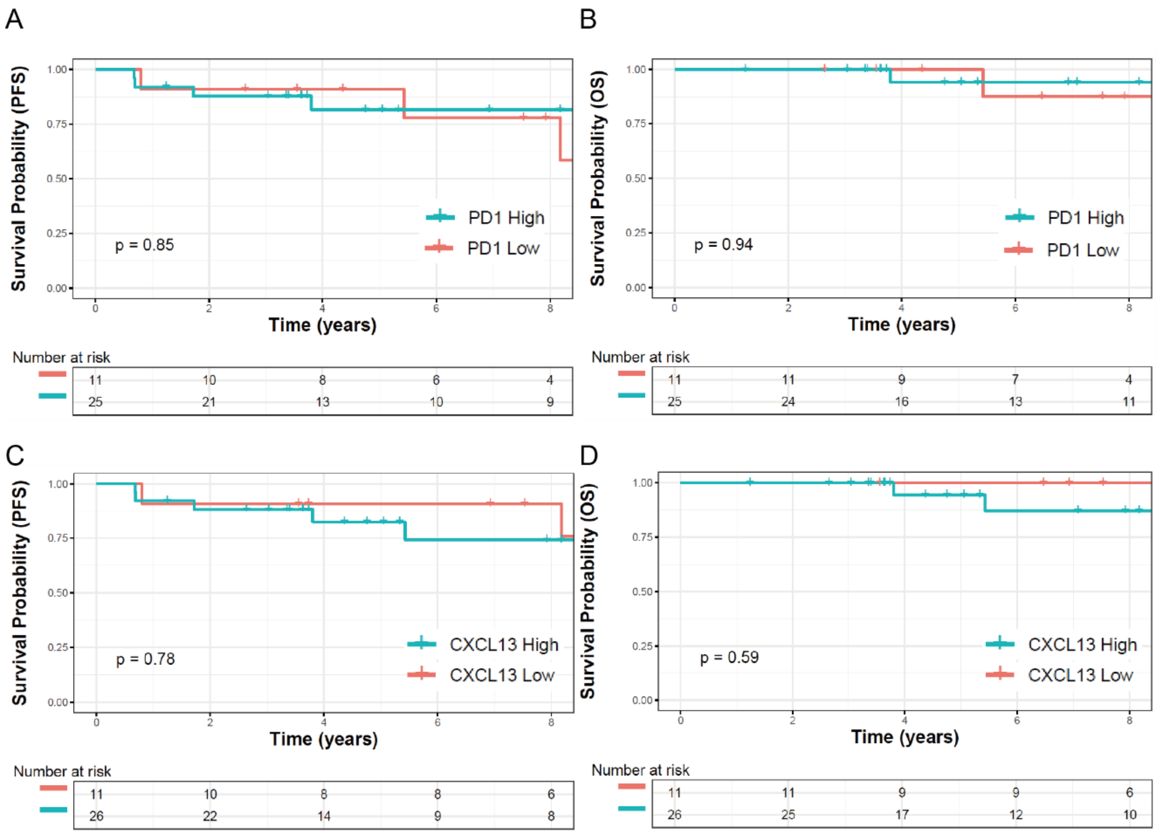

Supplemental Figure 9

Fig. S9. Survival analysis based on PD-1^{+} and CXCL13^{+} T cell proportions in LR-CHL. (*A-B*) The Kaplan-Meier survival curves of LR-CHL patients according to PD-1^{+} status for progression-free survival (*A*) and overall survival (*B*). (*C-D*) The Kaplan-Meier survival curves of LR-CHL patients according to CXCL13^{+} status for progression-free survival (*C*) and overall survival (*D*).

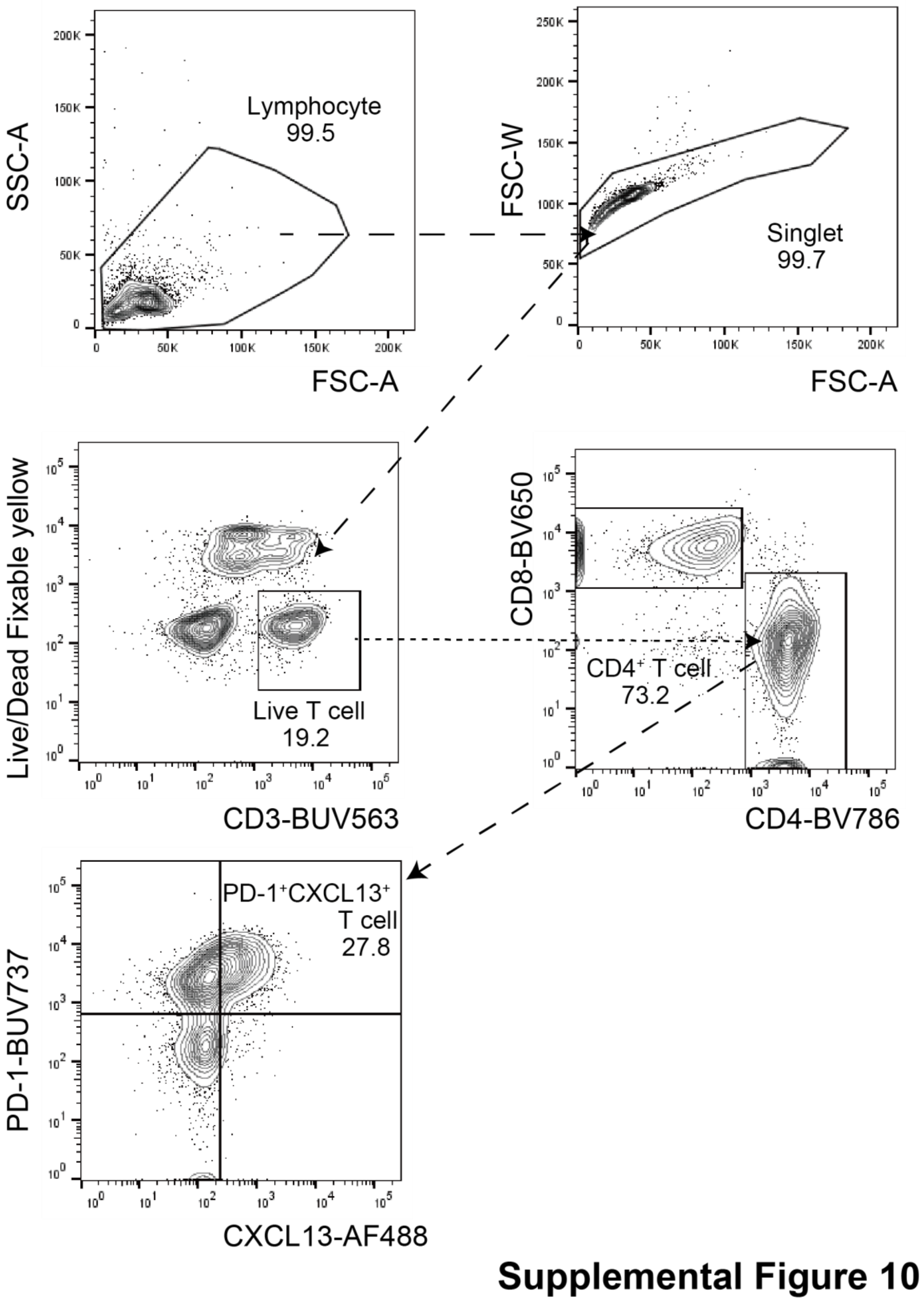

Fig. S10. Gating strategy used to assess CXCL13 and PD-1 expression on CD4$^+$ T cells (CD3$^+$CD4$^+$).

Table S1. Patient characteristics of the CHL study cohort for single cell RNA sequencing. EBV: Epstein-Barr virus

		All		Nodular sclerosis		Mixed cellularity		Lymphocyte rich	
Characteristic		No.	%	No.	%	No.	%	No.	%
Patient number		28		11		9		8	
Age at diagnosis (years)									
	Median	46		39		35		52	
	Range	20-75		26-67		20-75		29-59	
	>60 years	4	14	2	18	2	18	0	0
Gender, Male		21	75	7	64	8	89	6	75
Advanced Stage		9	32	5	45	2	25	2	25
IPS (International Prognostic Score) ≥4		4	14	1	9	1	11	2	25
Tumor size									
	Median (cm)	4		5		4		3	
	≥10	3	11	2	18	1	11	0	0
EBV status positive		6	21	0	0	5	56	1	13

Table S2. Demographics and clinical and phenotypic characteristics of the CHL study cohort for single cell RNA sequencing.
CHL: classic Hodgkin lymphoma; IPS: International Prognostic Score; EBV: Epstein-Barr Virus.

Case	Age	Gender	Disease Subtype	Tumor Size	Stage	IPS	EBV Infection
CHL02	28	Male	Mixed Cellularity	4	2A	Not available	Positive
CHL03	39	Male	Nodular Sclerosis	2	3B	Not available	Negative
CHL04	51	Male	Mixed Cellularity	2	1A	2	Positive
CHL05	67	Female	Nodular Sclerosis	Not available	3A	Not available	Negative
CHL06	64	Male	Nodular Sclerosis	5	1A	Not available	Negative
CHL07	29	Female	Nodular Sclerosis	4	3A	1	Negative
CHL08	26	Male	Nodular Sclerosis	5	2B	1	Negative
CHL09	55	Female	Nodular Sclerosis	3	3A	1	Negative
CHL10	29	Female	Nodular Sclerosis	10	2B	3	Negative
CHL11	22	Male	Mixed Cellularity	4	2A	1	Positive
CHL12	35	Male	Mixed Cellularity	4	4B	4	Positive
CHL13	31	Male	Mixed Cellularity	5	3B	1	Positive
CHL14	53	Male	Mixed Cellularity	4	2A	2	Negative
CHL15	20	Female	Mixed Cellularity	10	2A	2	Negative
CHL16	56	Male	Nodular Sclerosis	5	2A	Not available	Negative
CHL17	29	Male	Nodular Sclerosis	15	2A	1	Negative
CHL18	66	Male	Mixed Cellularity	Not available	2B	3	Negative
CHL19	33	Male	Nodular Sclerosis	8	2A	Not available	Negative
CHL20	75	Male	Mixed Cellularity	3	Unstaged	Not available	Negative
CHL21	41	Male	Lymphocyte rich	8	4B	4	Negative
CHL22	56	Male	Nodular Sclerosis	3	3A	4	Negative
CHL23	59	Female	Lymphocyte rich	2	1A	0	Negative
CHL24	56	Male	Lymphocyte rich	2	1A	2	Negative
CHL25	41	Female	Lymphocyte rich	2	2B	0	Negative
CHL26	56	Male	Lymphocyte rich	4	2B	2	Negative
CHL27	52	Male	Lymphocyte rich	2	2A	2	Not available
CHL28	29	Male	Lymphocyte rich	5	4B	4	Negative
CHL29	52	Male	Lymphocyte rich	4	1A	2	Positive

Table S3. Sequencing metrics for the scRNA-seq cohort as determined by CellRanger. CHL: classic Hodgkin lymphoma; RLN: reactive lymph node.

Case	Diagnosis	Batch info	Targeted Cell Recovery	Cells Sequenced	Mean Reads Per Cell	Sequencing Saturation (%)	Filtered cells	Median Genes Per Cell (filtered)
CHL02	HL	CHIP3	5000	3099	28400	70.5	3060	1018
CHL03	HL	CHIP3	5000	5635	26906	70.9	5511	1095
CHL04	HL	CHIP5	5000	4490	29348	70.9	4342	1143
CHL05	HL	CHIP4	5000	4299	33276	72.2	4220	1272
CHL06	HL	CHIP4	5000	4411	29254	71.4	4295	1210
CHL07	HL	CHIP3	5000	5773	20457	62.1	5600	1041
CHL08	HL	CHIP2	5000	2756	48981	81.6	2734	1119
CHL09	HL	CHIP2	5000	4689	32889	77	4637	1113
CHL10	HL	CHIP5	5000	7216	19984	54.3	7150	1341
CHL11	HL	CHIP5	5000	4868	23096	65.9	4738	1110
CHL12	HL	CHIP3	5000	6972	16269	57.4	6686	1066
CHL13	HL	CHIP2	5000	4186	30651	73.4	4097	1155
CHL14	HL	CHIP4	5000	4454	29885	73.5	4367	1217
CHL15	HL	CHIP4	5000	3758	33830	74.5	3667	1213
CHL16	HL	CHIP6	5000	3558	43560	76.5	3430	1229
CHL17	HL	CHIP6	5000	5568	28260	67.1	5489	1354
CHL18	HL	CHIP6	5000	4426	34292	68.3	4359	1522
CHL19	HL	CHIP6	5000	5935	26345	66.5	5902	1316.5
CHL20	HL	CHIP7	5000	3561	43153	78.9	3444	1236.5
CHL21	HL	CHIP7	5000	4856	29832	73.7	4764	1224
CHL22	HL	CHIP7	5000	6443	26841	71.2	6295	1140
CHL23	HL	CHIP8	5000	3423	45194	82.6	3287	1162
CHL24	HL	CHIP8	5000	2778	54975	84.5	2644	1252
CHL25	HL	CHIP8	5000	3313	47083	82.7	3134	1110.5
CHL26	HL	CHIP8	5000	3074	47972	86.6	2939	918
CHL27	HL	CHIP9	5000	4283	34031	78.4	4177	1023
CHL28	HL	CHIP9	5000	3818	37552	75.8	3649	1208
CHL29	HL	CHIP9	5000	4539	33246	75.5	4446	1065
RLN-1_R1	RLN	CHIP1	5000	4582	31271	73.4	4422	1152.5
RLN-1_R2	RLN	CHIP1	5000	4709	33504	75.6	4509	1132
RLN-2	RLN	CHIP9	5000	4119	37843	76.1	3956	1195
RLN-3	RLN	CHIP9	5000	3493	40683	77	3366	1260
RLN-4	RLN	CHIP9	5000	3802	35345	69.8	3629	1366
RLN-5	RLN	CHIP9	5000	3725	37604	72.6	3492	1279

Table S4. Demographics and clinical and phenotypic characteristics of the LR-CHL tissue microarray cohort.
LRCHL: Lymphocyte rich classic Hodgkin lymphoma; EBV: Epstein-Barr Virus.

Case	Age	Gender	Disease Subtype	Stage	EBV Infection
LRCHL01	63	Male	Lymphocyte rich	3A	Negative
LRCHL02	38	Male	Lymphocyte rich	1A	Negative
LRCHL03	61	Male	Lymphocyte rich	1A	Negative
LRCHL04	62	Female	Lymphocyte rich	2A	Negative
LRCHL05	51	Male	Lymphocyte rich	2A	Negative
LRCHL06	34	Female	Lymphocyte rich	3B	Negative
LRCHL07	24	Male	Lymphocyte rich	2A	Negative
LRCHL08	87	Female	Lymphocyte rich	2B	Negative
LRCHL09	70	Female	Lymphocyte rich	2A	Negative
LRCHL10	24	Male	Lymphocyte rich	4A	Negative
LRCHL11	79	Male	Lymphocyte rich	2B	Negative
LRCHL12	63	Male	Lymphocyte rich	1A	Negative
LRCHL13	47	Male	Lymphocyte rich	2A	Negative
LRCHL14	78	Female	Lymphocyte rich	2A	Negative
LRCHL15	67	Male	Lymphocyte rich	1A	Positive
LRCHL16	47	Male	Lymphocyte rich	3A	Positive
LRCHL17	57	Male	Lymphocyte rich	3B	Positive
LRCHL18	50	Male	Lymphocyte rich	3A	Negative
LRCHL19	69	Male	Lymphocyte rich	2A	Negative
LRCHL20	44	Male	Lymphocyte rich	1A	Negative
LRCHL21	55	Male	Lymphocyte rich	1A	Positive
LRCHL22	61	Female	Lymphocyte rich	2A	Negative
LRCHL23	46	Male	Lymphocyte rich	4A	Negative
LRCHL24	14	Female	Lymphocyte rich	NA	Positive
LRCHL25	40	Male	Lymphocyte rich	2A	Negative
LRCHL26	58	Male	Lymphocyte rich	1A	Positive
LRCHL27	70	Male	Lymphocyte rich	1A	Negative
LRCHL28	62	Male	Lymphocyte rich	1A	Negative
LRCHL29	81	Male	Lymphocyte rich	2A	Negative
LRCHL30	71	Female	Lymphocyte rich	4A	Negative
LRCHL31	38	Male	Lymphocyte rich	1A	Negative

Table S5. Patient characteristics of the entire LR-CHL cohort for survival analyses. LR-CHL: lymphocyte rich classic Hodgkin lymphoma, EBV: Epstein-Barr virus.

Characteristic		All		CD4/PD-1/CXCL13 High		CD4/PD-1/CXCL13 Low		P-value
		No.	%	No.	%	No.	%	
Patient number		37		12		25		
Age at diagnosis (years)								
	Median	56		54		56		
	Range	14-81		24-81		14-79		
	>60 years	14	38	5	42	9	36	0.28
Gender, Male		23	62	6	50	17	68	0.47
Advanced Stage		13	35	5	42	8	32	0.72
IPS (International Prognostic Score) ≥4		3	8	1	8	2	8	> 0.99
Tumor size								
	Median (cm)	4						
	≥10	0	0	0	0	0	0	> 0.99
EBV status positive		6	16	2	17	4	16	> 0.99

Table S6. Univariate analyses of prognostic factors' effect on progression-free survival (PFS) in LR-CHL patients (N = 37).

Clinical feature	P-value for PFS
Age ≥ 45	0.28
Sex, Male	0.86
Advanced Stage	0.24
IPS	0.40
EBV, positive	0.96
CXCL13	0.78
PD1	0.85
CD4/PD-1/CXCL13 High	0.04

Table S7. Antibodies used for immunohistochemistry staining.

Antigen	Antibody Clone	Manufacturer	Catalog number
CD30	CON6D/B5	Biocare	CM346
CD4	EPR6855	Abcam	ab133616
CXCL13	53610	R & D system	MAB801
CXCR5	EPR23463-30	Abcam	ab254415
PD-1	MRQ22	Cell Marque	315M
PD-1	NAT105	Cell Marque	315M-94
CD20	L26	Dako	M0755
CD20	L26	Bio Care Medical	CM004
PD-L1	SP142	Abcam	ab228462
TGF-b	EPR21143	Abcam	ab215715
BCL6	LN22	Bio Care Medical	CM410A

Table S8. Antibodies used for flow cytometry.

Antigen	Fluorescence	Antibody Clone	Manufacturer	Catalog number	Lot number
CD3	BUV563	SK7	BD Biosciences	741448	9233566
CD4	BV786	SK3	BD Biosciences	563877	8353751
CD8	BV650	RPA-T8	BD Biosciences	563821	9073878
CXCR5	APC-Cy7	J252D4	BioLegend	356926	B290954
CXCL13	AF488	53610	R & D System	IC801G	1554733
PD-1	BUV737	EH12.1	BD Biosciences	612791	9212298
ICOS	PE-Cyanine7	ISA-3	Invitrogen	25-9948-42	1989135
BCL-6	BV421	K112-91	BD Biosciences	563363	9322764
CD20	PE-Cyanine7	B9E9	Beckman Coulter	IM3629	200068
